# Supplementary material for: Predicting T cell receptor functionality against mutant epitopes
Source: Cell Genom. 2024 Aug 15;4(9):100634. doi: 10.1016/j.xgen.2024.100634 (PMC11480844; doi:10.1016/j.xgen.2024.100634)
Supplement: Document S1. Figures S1–S20 and Tables S1–S3 [file mmc1.pdf]

**Cell Genomics, Volume *4***

## Supplemental information

# Predicting T cell receptor functionality against mutant epitopes

**Felix Drost, Emilio Dorigatti, Adrian Straub, Philipp Hilgendorf, Karolin I. Wagner, Kersten Heyer, Marta López Montes, Bernd Bischl, Dirk H. Busch, Kilian Schober, and Benjamin Schubert**

# Supplementary Figures

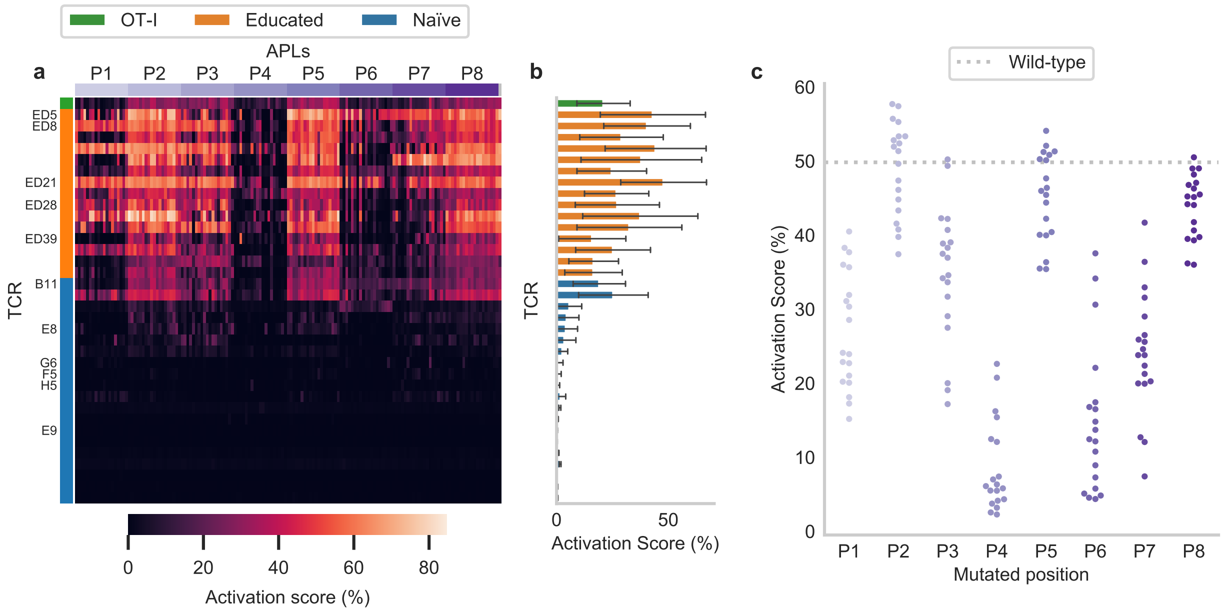


**Figure S1 | Unnormalized T cell activation for the TCRs and APLs of the murine dataset, related to Figure 2.** **a**, Unnormalized activation scores. **b**, Unnormalized activation scores averaged for all APLs and the wildtype epitope ($n = 153$) per TCR. **c**, Unnormalized activation per APL ($n = 19$) over all TCRs ($n = 36$).

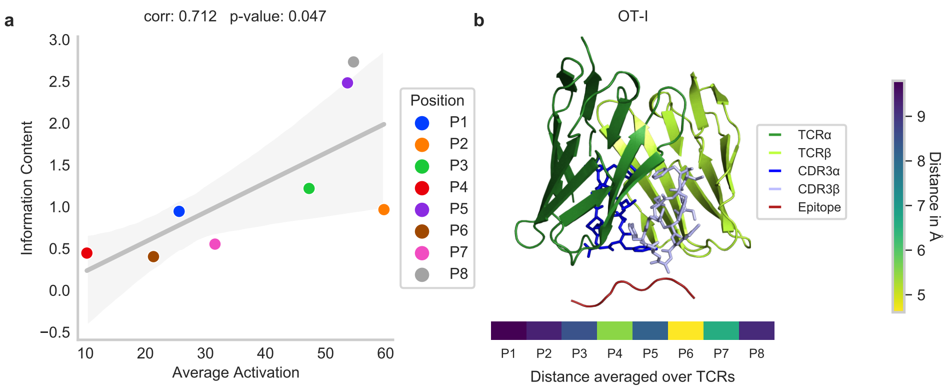


**Figure S2 | Influence of MHC restrictions on the activation score, related to Figure 2.** **a**, Correlation between the information content of the MHC motif positions measured in bits with the average activation scores for APLs mutated at these positions ($n = 8$) for H-2K$^b$. **b**, Structural model of OT-I/SIINFEKL predicted by TCRpMHCmodels [S1] and the distance of the residue to the nearest residue in the TCR averaged across the TCRs ($n = 32$) of the murine dataset.

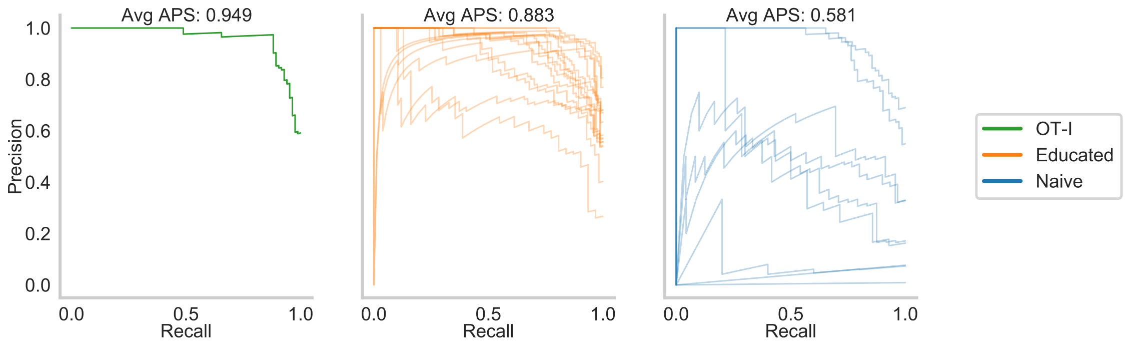


**Figure S3 | Additional metrics for predicting within a TCR of the murine dataset, related to Figure 2.** Precision-Recall curve with Average Precision Scores (APS) for the different groups of TCRs.

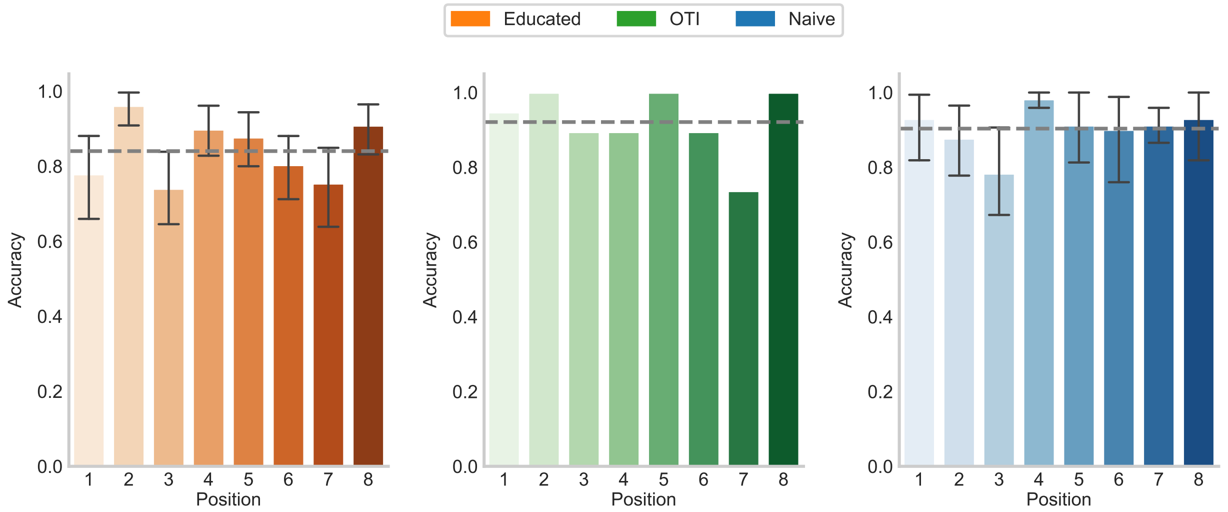


**Figure S4 | Position-wise evaluation, related to Figure 2.** Accuracy evaluated per position of mutation for the educated repertoire ($n = 15$), OT-I ($n = 1$), and the naive repertoire ($n = 9$) of the murine dataset. The dashed line shows the average position-wise accuracy over the whole dataset.

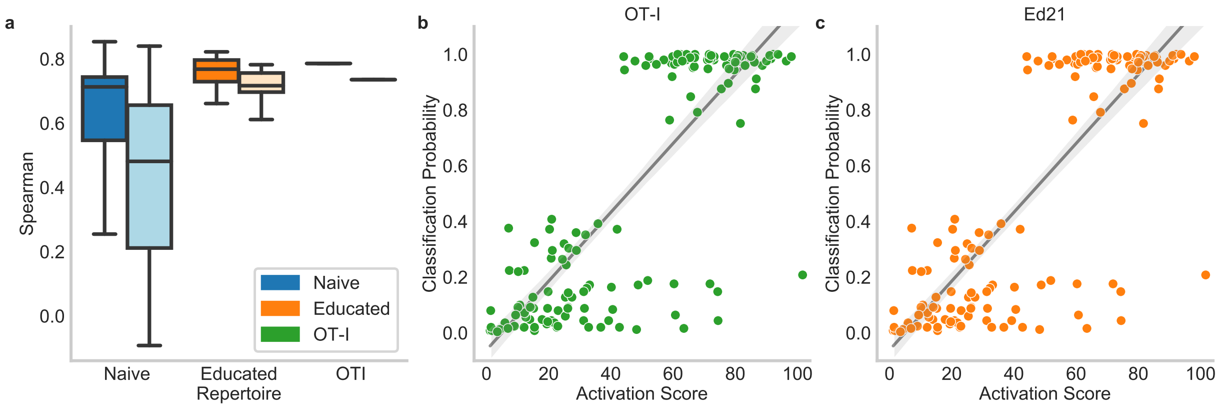


**Figure S5 | Regression capacity of the classification model for the murine datasets, related to Figure 2.** **a**, Regression evaluation of the regression model (dark color) compared to the classification model (light color) for the educated repertoire ($n = 15$), OT-I ($n = 1$), and the naive repertoire ($n = 9$) of the murine dataset. Exemplary correlation between activation score and classification probability of two representative TCRs: OT-I (**b**) and Ed21 (**c**) ($n = 152$ APLs).

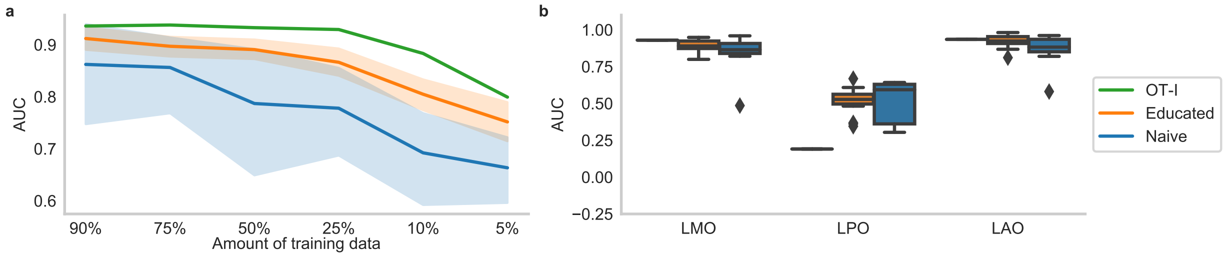


**Figure S6 | Additional metrics for reducing the training data within a TCR of the murine dataset, related to Figure 2. a**, AUC obtained when training on different subsets of the data. LMO: leave-mutation-out, LPO: leave-position-out, and LAO: leave-amino-acid-out. **b**, AUC when a smaller amount of training data is used (average over ten repetitions with random subsets for each TCR). The performance is shown for OT-I ($n = 1$ TCR), the educated repertoire ($n = 15$ TCRs), and the naïve repertoire ($n = 9$ TCRs).

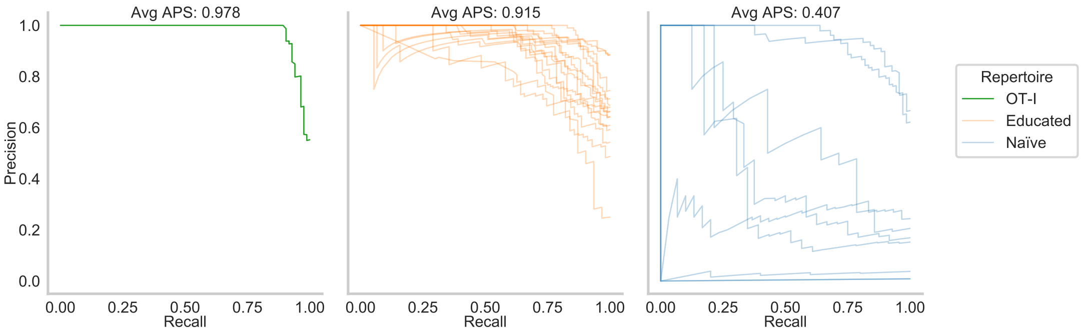


**Figure S7 | Additional metrics for predicting across TCRs of the murine dataset, related to Figure 3.** Precision-Recall curve with Average Precision Score (APS) for the different groups of TCRs.

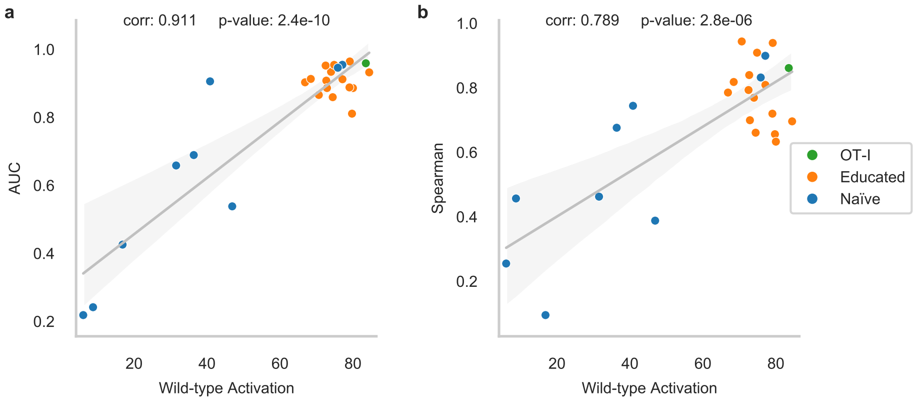


**Figure S8 | Relationship between generalization performance and wild-type activation, related to Figure 3.** The performance for classification (**a**) and regression (**b**) in the Leave-TCR-out setting shows a strong Pearson correlation to the normalized activation score against the wild-type epitope in the murine dataset ($n = 25$ TCRs).

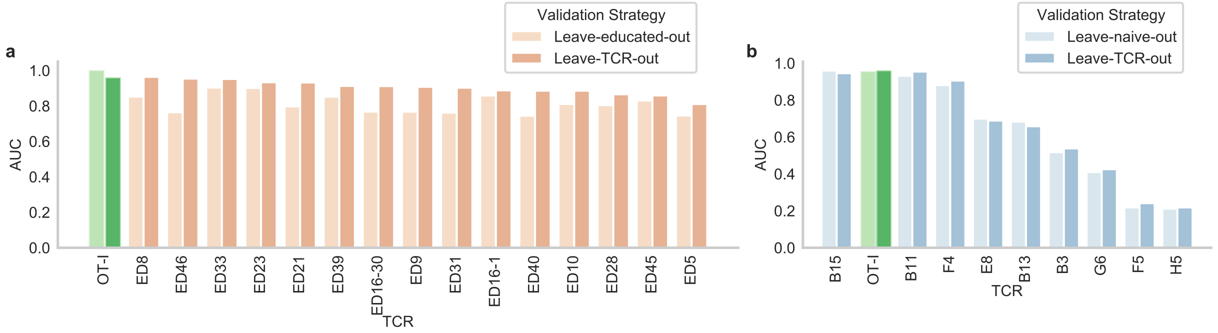


**Figure S9 | Comparison of the AUC scores for training on different repertoires, related to Figure 3.** **a**, Performance was evaluated on the 15 TCRs from the educated repertoire and OT-I when the model was trained on the full remaining murine dataset (Leave-TCR-out) or solely the TCRs of the naive repertoire (Leave-educated-out). **b**, Performance on the nine TCRs from the naive repertoire and OT-I for Leave-TCR-out training or training only on the TCRs from the educated repertoire (Leave-naive-out).

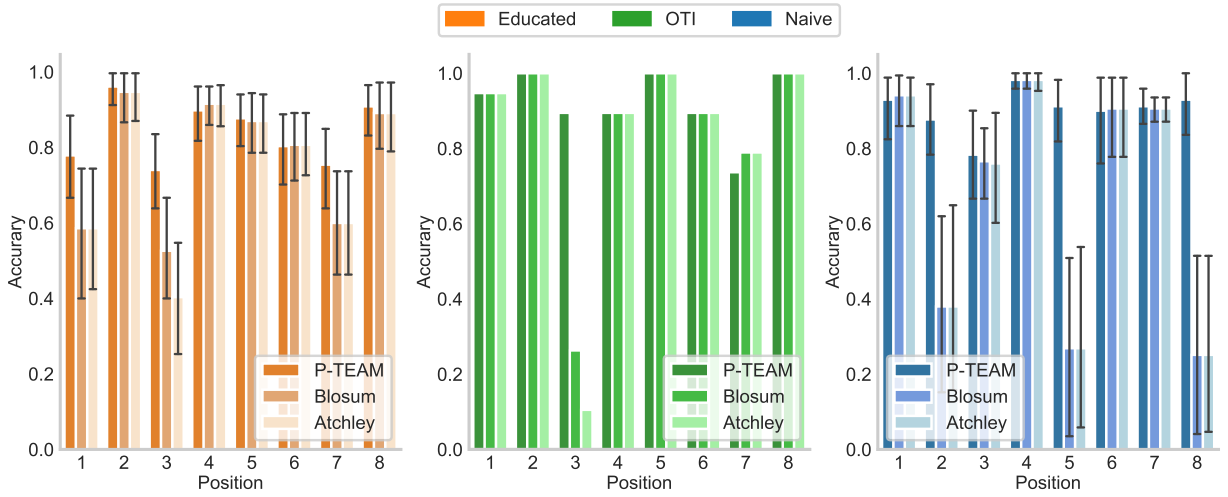


**Figure S10 | Position-wise evaluation compared to peptide distances, related to Figure 3.** Accuracy of P-TEAM evaluated per position of mutation compared to the amino acid distances of BLOSUM62 and Atchley factors for the educated repertoire ($n = 15$), OT-I ($n = 1$), and the naive repertoire ($n = 9$) of the murine dataset.

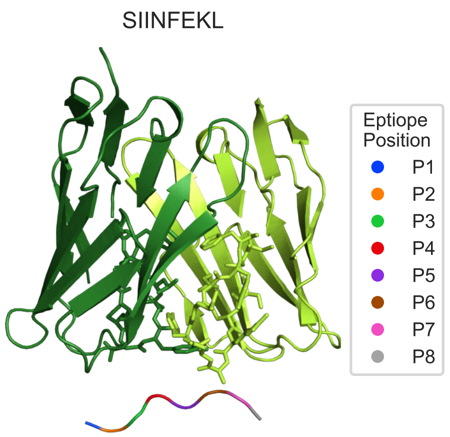


**Figure S11 | Epitope positions in the structural model, related to Figure 3.** Epitope positions are highlighted in the structural model for OT-I/SIINFEKL. TCR$\alpha$ and TCR$\beta$ chains are shown in dark and light green, respectively.

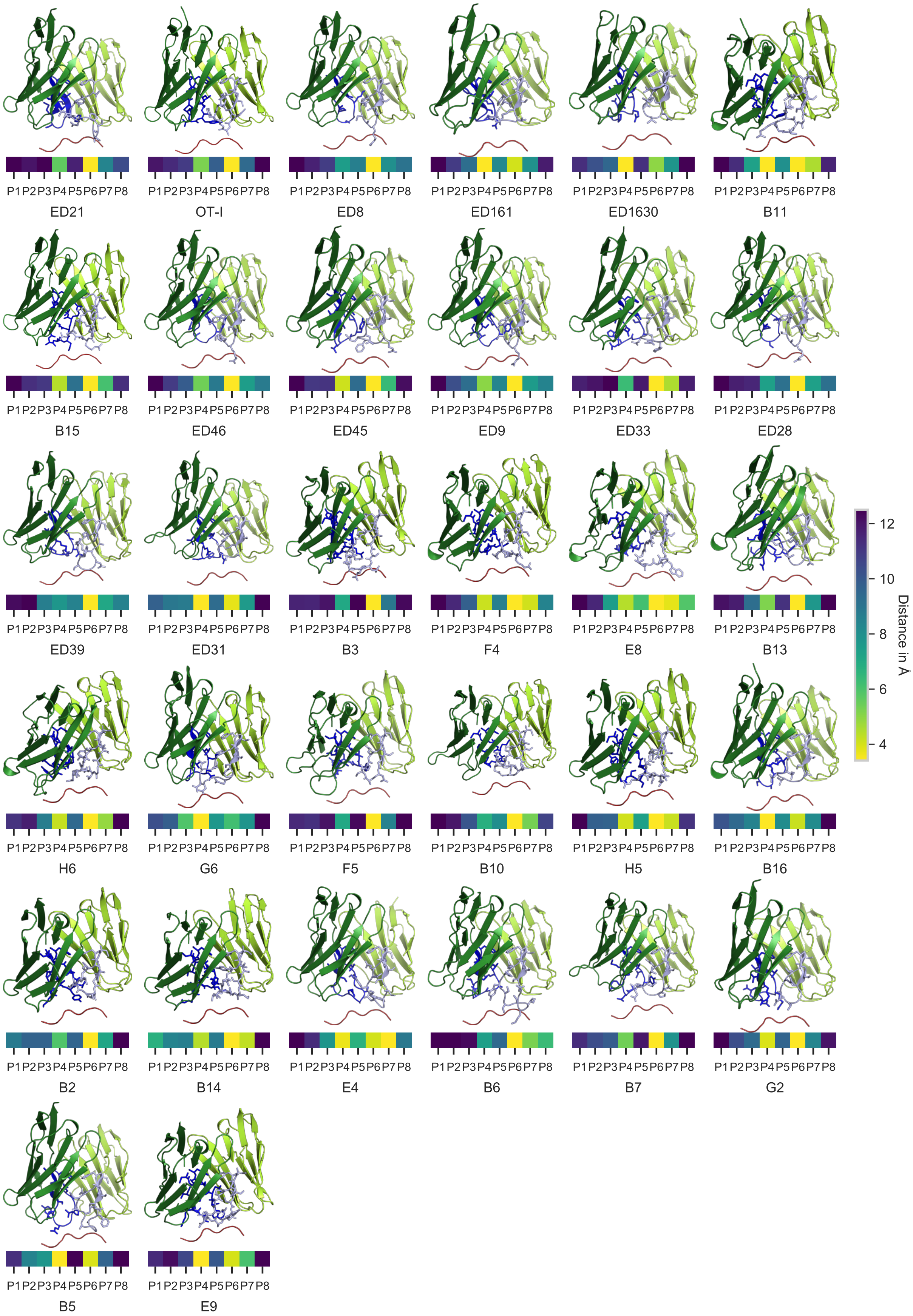


**Figure S12 | Structural Models of the murine dataset, related to Figure 3.** Predicted structures of the TCR and epitope, and minimal distance to the individual epitope positions for all receptors of the murine datasets ordered by descending activation to the wildtype epitope.

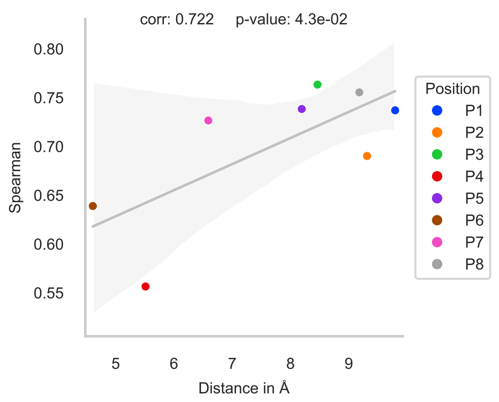


**Figure S13 | Relationship between spacial distance and feature importance, related to Figure 3.** The regression performance during perturbation tests at each epitope position shows strong Pearson correlation to the distance between this position and its closest TCR residue in the murine dataset ($n$ = 8 positions).

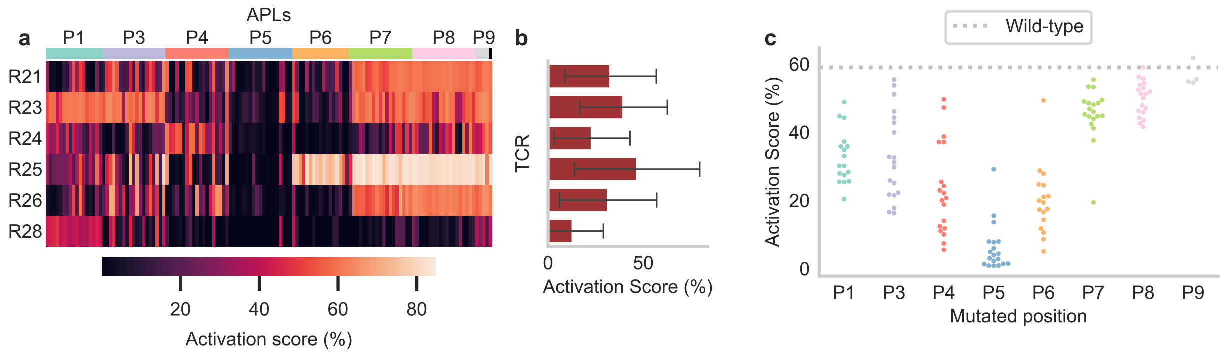


**Figure S14 | Unnormalized T cell activation for the TCRs and APLs of the neo-epitope dataset, related to Figure 5. a**, Unnormalized activation scores. **b**, Unnormalized activation scores averaged for all APLs ($n = 133$) per TCR. **c**, Unnormalized activation per APL over all TCRs ($n = 6$).

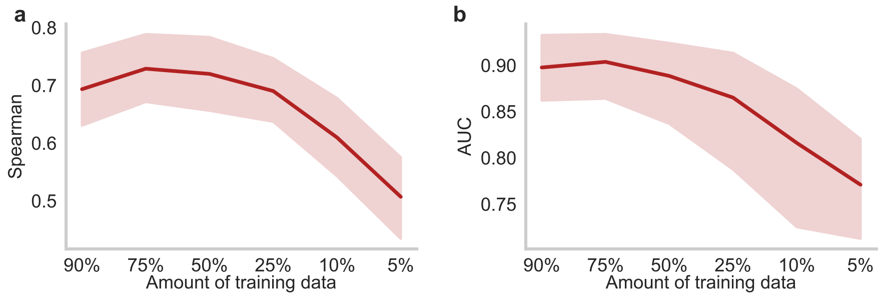


**Figure S15 | Performance on the human dataset on limited training data, related to Figure 5.** Spearman correlation (**a**) and AUC (**b**) when a smaller amount of training data is used ($n = 6$ TCRs $*$ 100 repetitions).

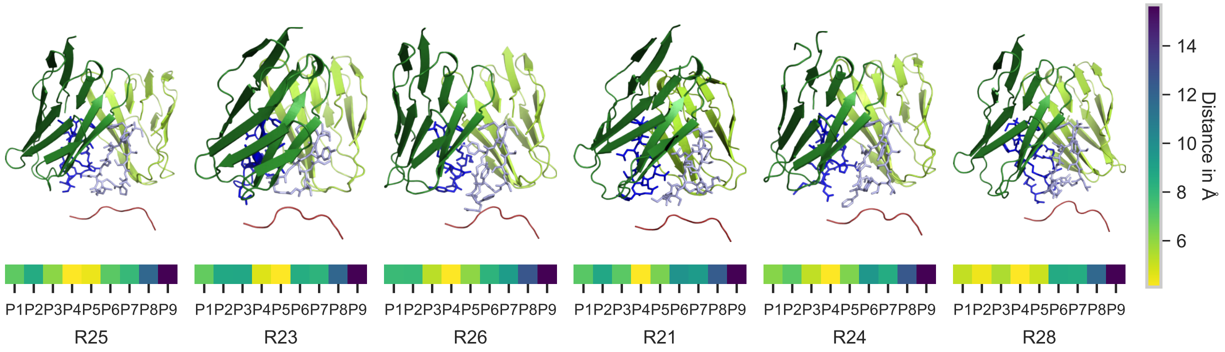


**Figure S16 | Structural Models of the neo-epitope dataset, related to Figure 6.** Predicted structures of the TCR and epitope, and minimal distance to the individual epitope positions for all receptors of the human datasets ordered by descending activation to the wildtype epitope.

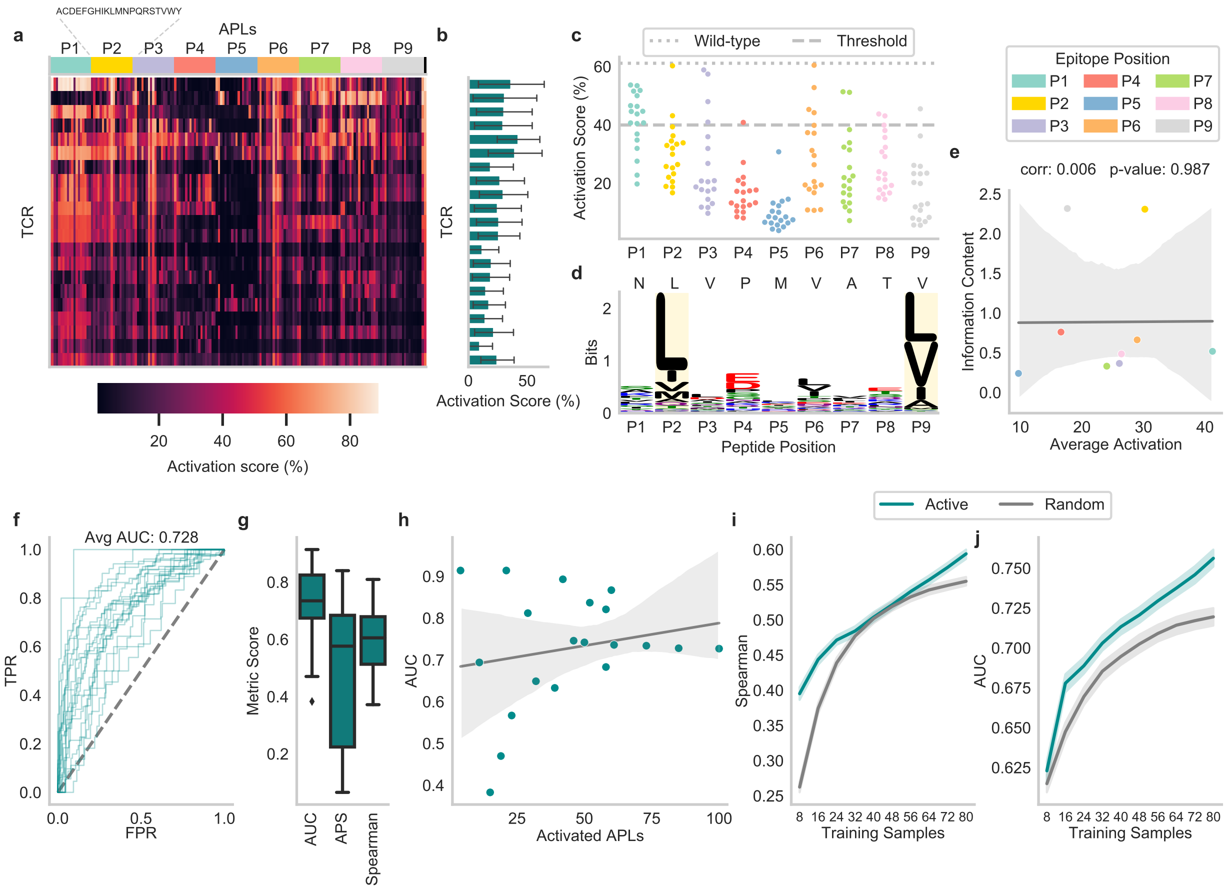


**Figure S17 | Predicting the effect of mutations of a CMV epitope, related to Figure 2 and Figure 4.** **a**, The normalized activation scores of 20 TCRs of the CMV dataset. **b**, The activation scores averaged for all APLs ($n = 171$) of one TCR. **c**, The epitope position on which the mutation occurs strongly influences the activation per APL ($n = 19$) averaged over all TCRs ($n = 20$). The threshold value represents the boundary between binding and non-binding and wild-type indicates the activation scores of the base epitope NLVPMVATV. **d**, MHC restrictiveness indicated by information content in bits for HLA-A*02:01 obtained from the MHC Motif Atlas [S2] per position from $n = 8,372$ peptides. Reported anchor positions are highlighted in yellow and the wildtype epitope is indicated above. **e**, Correlation between the Gini coefficient of the MHC motif positions with the average activation scores for APLs mutated at these positions for HLA-A*02:01 ($n = 9$ positions) **f**, The Receiver operating characteristic (ROC) curves of the CMV TCRs indicate the True Positive Rate (TPR) against the False Positive Rate (FPR) at all prediction values as thresholds. **g**, Different evaluation metrics for regression (Spearman) and classification models (APS: Average Precision Score, AUC: Area Under the ROC Curve). **h**, Performance of P-TEAM against the amount of activated APLs per TCR. The performance in e-g is shown for $n = 20$ TCRs. Comparison of the active learning framework to random sample selection on the CMV dataset for regression (**i**) and classification models (**j**). The expected performance is shown for up to $m = 10$ consecutive iterations ($N_{APLs} = 80$ APLs) of alternating wet lab experiments and model training ($n = 15$ TCRs $*$ 100 repetitions).

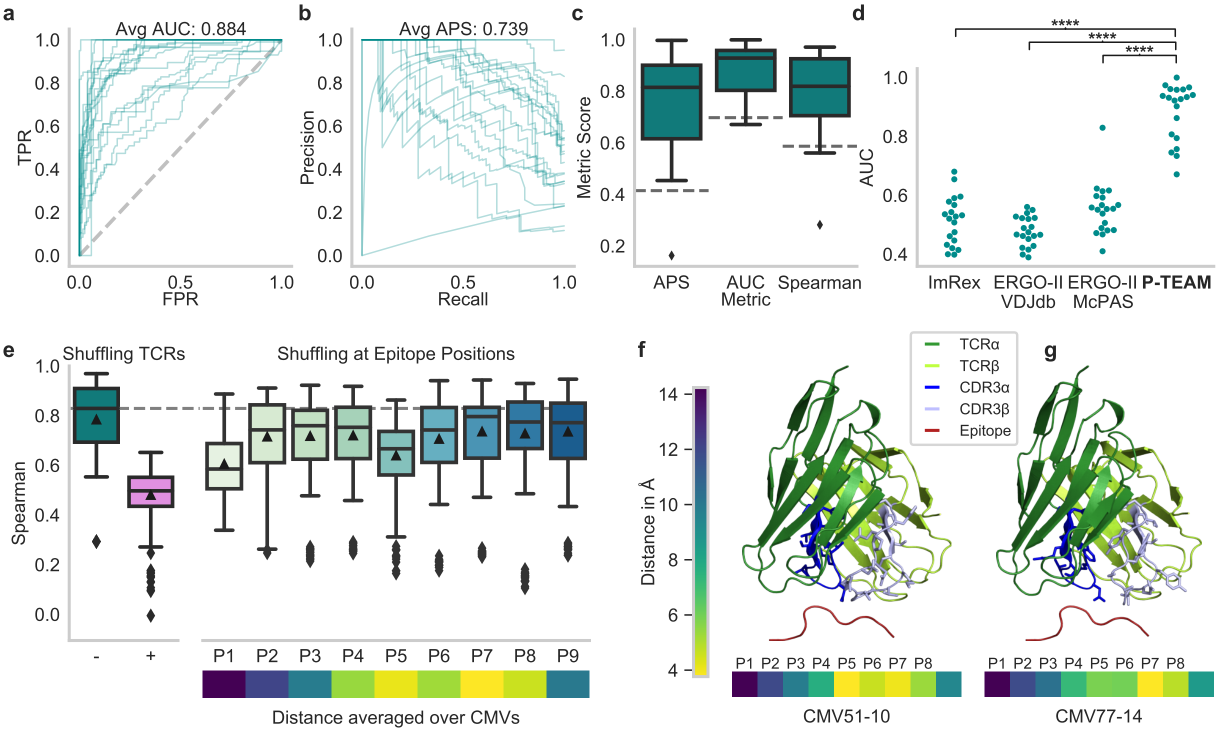


**Figure S18 | Across-repertoire prediction for the CMV dataset, related to Figure 3.** ROC (**a**) and Precision-Recall (**b**) curves for the 20 CMV-specific TCRs. **c**, Average precision score (APS), area under the ROC curve (AUC), and Spearman correlation as classification and regression metrics. The dashed line indicates the prediction using the labels of a random other TCR. **d**, P-TEAM outperforms existing TCR-epitope predictors ImRex [S3] and ERGO-II [S4] by a large margin (****: p-value $< 0.0001$). The performance in a-d is shown for $n = 20$ TCRs. **e**, The importance of input features obtained by replacing the test TCR input with a random CDR3 sequence of the dataset ($+$) or by shuffling the amino acid at each epitope position in the test set compared to the un-shuffled performance (- and dashed line) ($n = 20$ TCRs $*$ 15 repetitions). Below, the average distance of the center of mass between the epitope and TCR residues is shown. **f, g**, Predicted structural model of the TCR and epitope, and minimal distance to the individual epitope positions for receptors CMV51-10 and CMV77-14 (highest and lowest activation, respectively). The model shows the interaction between the epitope and the CDR3 of the TCRs.

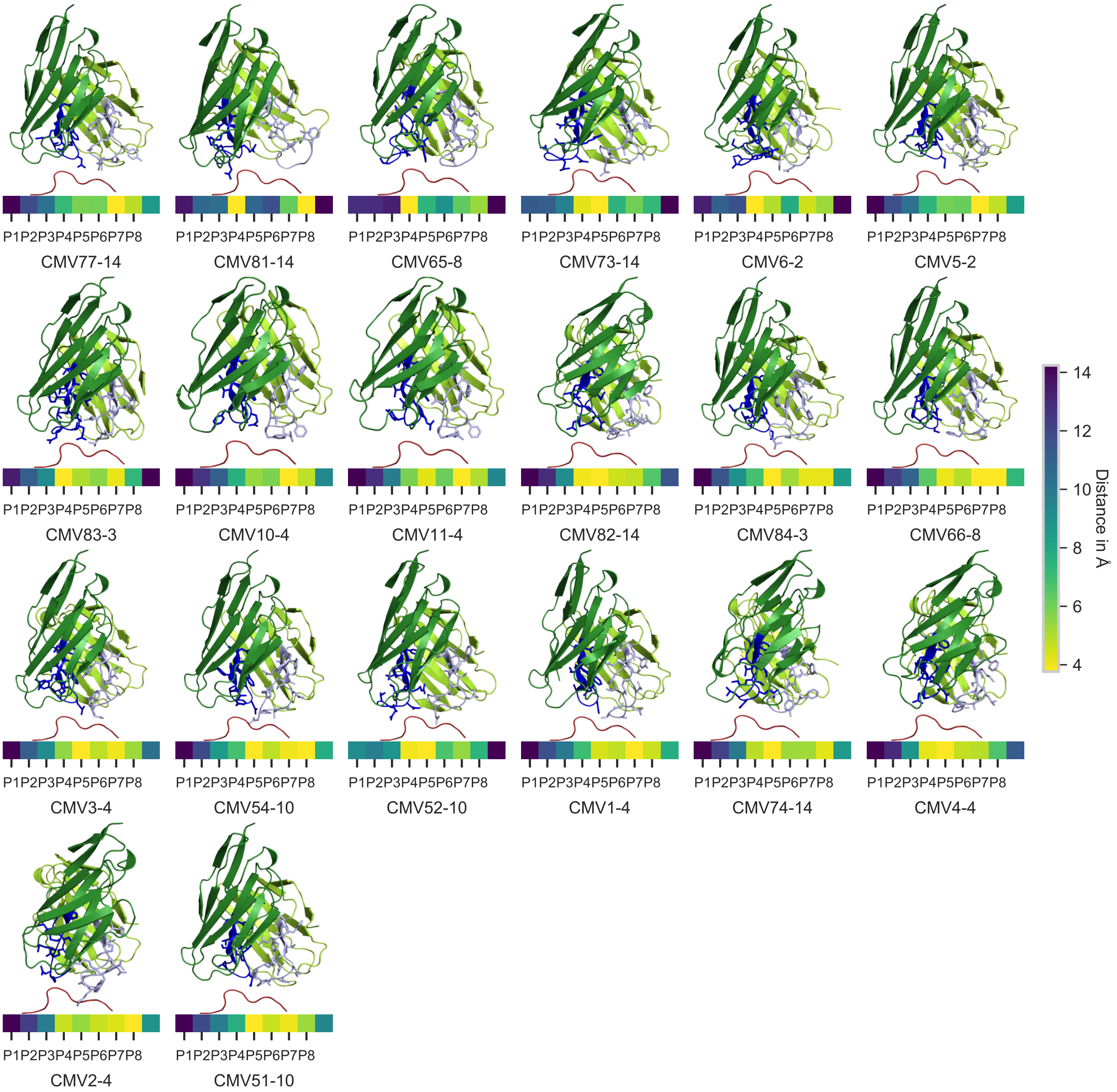


**Figure S19 | Structural Models of the CMV dataset, related to Figure 3.** Predicted structures of the TCR and epitope, and minimal distance to the individual epitope positions for all receptors of the human datasets ordered by descending activation to the wildtype epitope.

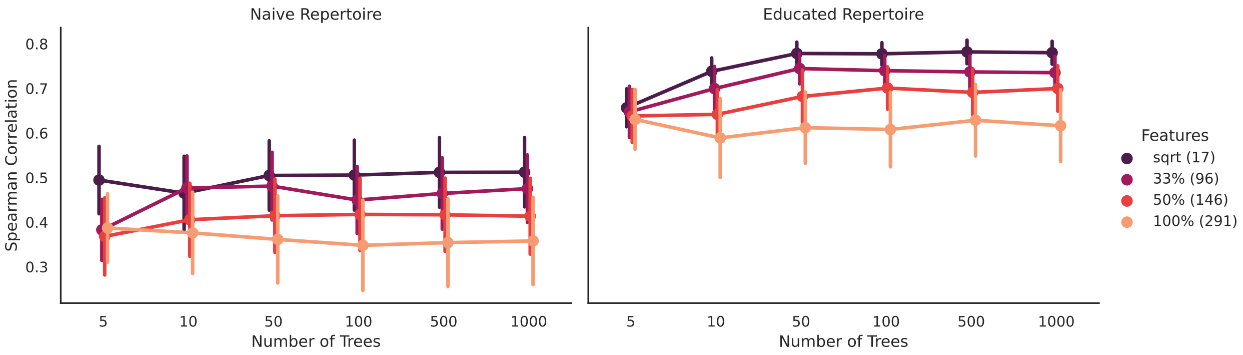


**Figure S20 | Performance of P-TEAM by number of trees in the random forest, and number of features used to build each tree, related to STAR Methods.** The Spearman correlation between the predicted and measured activation scores in a leave-TCR-out validation scheme is shown as a function of the number of trees used to train the random forests ($x$-axis), and the number of randomly-chosen features used to grow each tree.

## Supplementary Tables

| Repertoire | TCR | Leave-Mutation-Out AUC | APS | Spearman | Leave-TCR-out AUC | APS | Spearman |
|---|---|---|---|---|---|---|---|
| | OTI | 0.930 | 0.949 | 0.786 | **0.959** | **0.978** | 0.861 |
| Educated | ED5 | 0.907 | 0.946 | 0.746 | 0.811 | 0.880 | 0.656 |
| | ED8 | 0.938 | **0.980** | 0.724 | **0.965** | **0.990** | **0.939** |
| | ED9 | 0.875 | 0.900 | 0.772 | 0.908 | 0.938 | 0.840 |
| | ED10 | 0.905 | 0.930 | 0.755 | 0.886 | 0.945 | 0.633 |
| | ED16-1 | **0.944** | 0.946 | 0.802 | 0.888 | 0.928 | 0.720 |
| | ED16-30 | 0.920 | 0.941 | 0.801 | 0.912 | 0.925 | 0.809 |
| | ED21 | **0.949** | **0.987** | 0.709 | 0.933 | **0.982** | 0.696 |
| | ED23 | 0.891 | 0.949 | 0.661 | 0.934 | 0.973 | 0.769 |
| | ED28 | *0.799* | 0.668 | 0.735 | 0.866 | 0.807 | **0.944** |
| | ED31 | 0.873 | 0.804 | 0.793 | 0.903 | 0.859 | 0.785 |
| | ED33 | 0.928 | 0.908 | 0.708 | 0.953 | 0.923 | 0.793 |
| | ED39 | 0.873 | 0.662 | 0.768 | 0.913 | 0.805 | 0.818 |
| | ED40 | 0.850 | 0.829 | **0.822** | 0.886 | 0.895 | 0.700 |
| | ED45 | 0.841 | 0.912 | 0.777 | 0.860 | 0.919 | 0.661 |
| | ED46 | 0.890 | 0.880 | **0.822** | 0.955 | 0.960 | **0.909** |
| Naive | B3 | 0.919 | 0.602 | 0.739 | 0.538 | 0.379 | *0.388* |
| | B11 | **0.959** | **0.954** | **0.854** | **0.955** | 0.945 | 0.899 |
| | B13 | 0.858 | *0.521* | 0.713 | 0.659 | 0.275 | 0.462 |
| | B15 | 0.895 | 0.917 | 0.780 | 0.946 | 0.947 | 0.832 |
| | E8 | *0.821* | *0.447* | 0.665 | 0.689 | 0.456 | 0.676 |
| | F4 | 0.865 | 0.565 | 0.744 | 0.906 | 0.608 | 0.744 |
| | F5 | - | - | *0.547* | *0.242* | *0.009* | 0.457 |
| | G6 | *0.486* | *0.062* | *0.255* | *0.426* | *0.034* | *0.095* |
| | H5 | - | - | *0.307* | *0.219* | *0.008* | *0.255* |

**Table S1 | Performance of P-TEAM on the murine dataset, related to Figure 2 and Figure 3.** The classification performance is reported by the area under the receiver operator characteristic curve (AUC) and the average precision score (APS). The regression performance is reported by the Spearman's rank coefficient. The three TCRs on which P-TEAM performed best and worst are shown in bold and italics, respectively, for each metric. AUC and APS of F5 and H5 could not be calculated as no sample was predicted as positive.

| TCR | Leave-Mutation-Out AUC | APS | Spearman | Leave-TCR-out AUC | APS | Spearman |
|---|---|---|---|---|---|---|
| R21 | 0.906 | 0.882 | 0.682 | **0.938** | **0.916** | **0.800** |
| R23 | 0.892 | 0.872 | 0.744 | 0.788 | 0.842 | 0.708 |
| R24 | *0.761* | *0.322* | 0.700 | 0.618 | 0.275 | *0.427* |
| R25 | **0.949** | **0.933** | **0.846** | 0.809 | 0.864 | 0.681 |
| R26 | 0.920 | 0.901 | 0.780 | 0.916 | 0.899 | 0.766 |
| R28 | 0.911 | 0.791 | *0.654* | *0.561* | *0.183* | 0.509 |

**Table S2 | Performance of P-TEAM on the human dataset, related to Figure 5 and Figure 6.** The classification performance is reported by the area under the receiver operator characteristic curve (AUC) and the average precision score (APS). The regression performance is reported by the Spearman's rank coefficient. The TCRs on which P-TEAM performed best and worst are shown in bold and italics, respectively, for each metric.

| TCR | Leave-Mutation-Out AUC | APS | Spearman | Leave-TCR-out AUC | APS | Spearman |
|---|---|---|---|---|---|---|
| CMV4-4 | **0.914** | 0.780 | 0.775 | **1.000** | **0.998** | **0.971** |
| CMV51-10 | 0.914 | 0.116 | 0.643 | 0.940 | *0.163* | 0.719 |
| CMV82-14 | 0.893 | 0.709 | 0.775 | 0.757 | 0.707 | 0.959 |
| CMV65-8 | 0.866 | **0.840** | 0.699 | 0.734 | 0.584 | 0.666 |
| CMV84-3 | 0.838 | 0.672 | **0.810** | 0.927 | 0.871 | 0.899 |
| CMV81-14 | 0.821 | 0.676 | 0.658 | *0.671* | 0.566 | *0.282* |
| CMV54-10 | 0.812 | 0.556 | 0.616 | 0.937 | 0.801 | 0.830 |
| CMV66-8 | 0.746 | 0.597 | 0.628 | 0.958 | 0.902 | 0.863 |
| CMV10-4 | 0.742 | 0.540 | 0.595 | 0.974 | 0.916 | 0.966 |
| CMV11-4 | 0.736 | 0.633 | 0.557 | 0.961 | 0.954 | 0.959 |
| CMV77-14 | 0.734 | 0.655 | 0.692 | 0.922 | 0.901 | 0.853 |
| CMV5-2 | 0.728 | 0.770 | 0.514 | 0.902 | 0.903 | 0.809 |
| CMV6-2 | 0.727 | 0.796 | 0.373 | 0.794 | 0.846 | 0.632 |
| CMV3-4 | 0.694 | 0.109 | 0.675 | 0.966 | 0.627 | 0.782 |
| CMV73-14 | 0.683 | 0.437 | 0.526 | 0.931 | 0.893 | 0.931 |
| CMV52-10 | 0.649 | 0.251 | 0.512 | 0.959 | 0.830 | 0.925 |
| CMV2-4 | 0.633 | 0.331 | 0.540 | 0.862 | 0.710 | 0.798 |
| CMV83-3 | 0.567 | 0.144 | 0.388 | 0.933 | 0.676 | 0.735 |
| CMV74-14 | 0.470 | 0.098 | *0.372* | 0.745 | 0.454 | 0.561 |
| CMV1-4 | *0.383* | *0.065* | 0.495 | 0.807 | 0.485 | 0.610 |

**Table S3 | Performance of P-TEAM on the CMV dataset, related to Figure 2 and Figure 3.** The classification performance is reported by the area under the receiver operator characteristic curve (AUC) and the average precision score (APS). The regression performance is reported by the Spearman's rank coefficient. The TCRs on which P-TEAM performed best and worst are shown in bold and italics, respectively, for each metric.

# References


[S1] Jensen, K. *et al.* Tcrpmhcmodels: Structural modelling of tcr-pmhc class i complexes. *Scientific Reports* **9**, 1–12 (2019).

[S2] Tadros, D. M., Eggenschwiler, S., Racle, J. & Gfeller, D. The mhc motif atlas: a database of mhc binding specificities and ligands. *Nucleic Acids Research* **51**, D428–D437 (2023).

[S3] Moris, P. *et al.* Current challenges for unseen-epitope tcr interaction prediction and a new perspective derived from image classification. *Briefings in Bioinformatics* **22**, bbaa318 (2021).

[S4] Springer, I., Tickotsky, N. & Louzoun, Y. Contribution of t cell receptor alpha and beta cdr3, mhc typing, v and j genes to peptide binding prediction. *Frontiers in Immunology* **12**, 1436 (2021).
