# Supplementary material for: Predicting T cell receptor functionality against mutant epitopes
Source: Cell Genom. 2024 Aug 15;4(9):100634. doi: 10.1016/j.xgen.2024.100634 (PMC11480844; doi:10.1016/j.xgen.2024.100634)
Supplement: Document S2. Article plus supplemental information [file mmc11.pdf]

Cell Genomics

Article

Predicting T cell receptor functionality against mutant epitopes

Graphical abstract

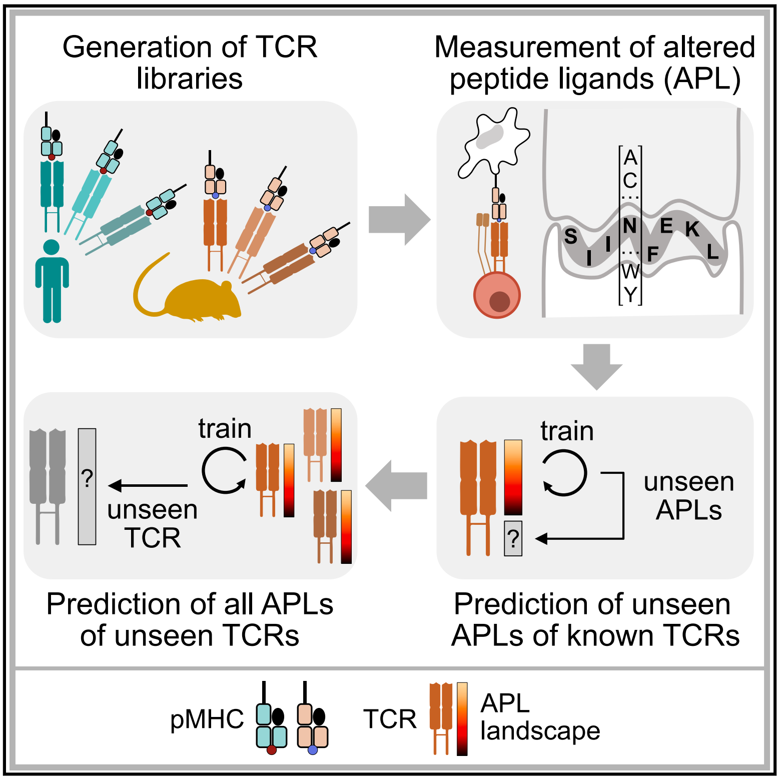

Authors

Felix Drost, Emilio Dorigatti, Adrian Straub, ..., Dirk H. Busch, Kilian Schober, Benjamin Schubert

Correspondence

benjamin.schubert@helmholtz-munich.de

In brief

Drost et al. present a machine learning model to predict the effect of mutations in antigen-derived epitopes on the T cell receptor activation landscape. The model was able to classify T cell reactivities and quantitatively predict T cell functionalities for unobserved mutations and unseen TCRs to identify cross-reactivity.

Highlights

- P-TEAM predicts the effect of epitope mutations on the T cell receptor (TCR) response
- The model was validated on murine and human datasets of systematic epitope mutations
- Predictions can be conducted for unseen mutations of the same TCR or novel TCRs
- P-TEAM estimates immune escape and cross-reactivities of therapeutic TCRs

Drost et al., 2024, Cell Genomics *4*, 100634
September 11, 2024 © 2024 The Authors. Published by Elsevier Inc.
https://doi.org/10.1016/j.xgen.2024.100634

CellPress

Cell Genomics

CellPress
OPEN ACCESS

Article

Predicting T cell receptor functionality against mutant epitopes

Felix Drost,[1,2,10] Emilio Dorigatti,[1,3,4,10] Adrian Straub,[5,10] Philipp Hilgendorf,[5,6] Karolin I. Wagner,[5] Kersten Heyer,[5] Marta López Montes,[5] Bernd Bischl,[3,4] Dirk H. Busch,[5,7,11] Kilian Schober,[5,6,8,11] and Benjamin Schubert[1,9,11,12,*]

[1]Institute of Computational Biology, Helmholtz Center Munich, 85764 Neuherberg, Germany
[2]School of Life Sciences Weihenstephan, Technical University of Munich, 85354 Freising, Germany
[3]Department of Statistics, Ludwig Maximilian Universität, 80539 Munich, Germany
[4]Munich Center for Machine Learning (MCML), Ludwig Maximilian Universität, 80538 Munich, Germany
[5]Institute for Medical Microbiology, Immunology, and Hygiene, Technical University of Munich, 81675 Munich, Germany
[6]Mikrobiologisches Institut–Klinische Mikrobiologie, Immunologie, und Hygiene, Universitätsklinikum Erlangen, Friedrich-Alexander-Universität Erlangen-Nürnberg, 91054 Erlangen, Germany
[7]German Center for Infection Research, Deutschen Zentrum für Infektionsforschung (DZIF), Partner Site Munich, 81675 Munich, Germany
[8]Medical Immunology Campus Erlangen, Friedrich-Alexander-Universität (FAU) Erlangen-Nürnberg, 91054 Erlangen, Germany
[9]School of Computation, Information, and Technology, Technical University of Munich, 85748 Garching bei München, Germany
[10]These authors contributed equally
[11]These authors contributed equally
[12]Lead contact
*Correspondence: benjamin.schubert@helmholtz-munich.de
https://doi.org/10.1016/j.xgen.2024.100634

SUMMARY

Cancer cells and pathogens can evade T cell receptors (TCRs) via mutations in immunogenic epitopes. TCR cross-reactivity (i.e., recognition of multiple epitopes with sequence similarities) can counteract such escape but may cause severe side effects in cell-based immunotherapies through targeting self-antigens. To predict the effect of epitope point mutations on T cell functionality, we here present the random forest-based model Predicting T Cell Epitope-Specific Activation against Mutant Versions (P-TEAM). P-TEAM was trained and tested on three datasets with TCR responses to single-amino-acid mutations of the model epitope SIINFEKL, the tumor neo-epitope VPSVWRSSL, and the human cytomegalovirus antigen NLVPMVATV, totaling 9,690 unique TCR-epitope interactions. P-TEAM was able to accurately classify T cell reactivities and quantitatively predict T cell functionalities for unobserved single-point mutations and unseen TCRs. Overall, P-TEAM provides an effective computational tool to study T cell responses against mutated epitopes.

INTRODUCTION

The T cell receptor (TCR)-mediated recognition of pathogen- or tumor-derived epitopes by T cells plays an essential role in the adaptive immune response. These epitopes are bound to the major histocompatibility complex (MHC) and interact with the complementarity-determining regions (CDRs) of the TCR. T cells whose TCR recognizes the epitope with sufficient affinity are activated and undergo clonal expansion and differentiation to form an immune response. The exchange of a single amino acid in the epitope may severely alter TCR binding behavior[1] and can lead to a 10-fold higher antigen sensitivity in immunoassays.[2] Furthermore, undetected cross-reactivity toward healthy cells may cause severe damage when developing T cell-based immunotherapies against neo-epitopes from tumor cells and must therefore be avoided.[3]

Computationally predicting binding between TCR and epitope remains challenging due to the immense sequence diversity. It has been estimated that there exist more than 10^{20} possible TCRs in nature and that every human harbors at least 10^7 different TCRs at any given time.[4] Publicly available, curated datasets with paired information on TCRs and their recognized epitopes allowed the creation of a variety of machine learning methods to predict TCR-epitope binding.[5–10] However, these data are not collected in a standardized manner in the context of deep mutational epitope scans. As of April 2023, for example, only 17 of the 152 single-amino acid mutated peptides for the model epitope SIINFEKL are provided in the Immune Epitope Database[11] and none in the VDJdb.[12] Therefore, current methods trained on such data are likely to fail when predicting the change in T cell activation introduced by most point mutations. Additionally, these databases, and thereby the predictors as well, typically simplify the TCR-epitope interaction to a binary event of binding or non-binding, even though epitopes activate T cells to various degrees, resulting in continuous changes in the phenotype and abundance of T cell populations during an immune response.[13] The dataset from a deep mutational scan introduced by Straub et al.[14] tackles both of these shortcomings

This is an open access article under the CC BY license (http://creativecommons.org/licenses/by/4.0/).

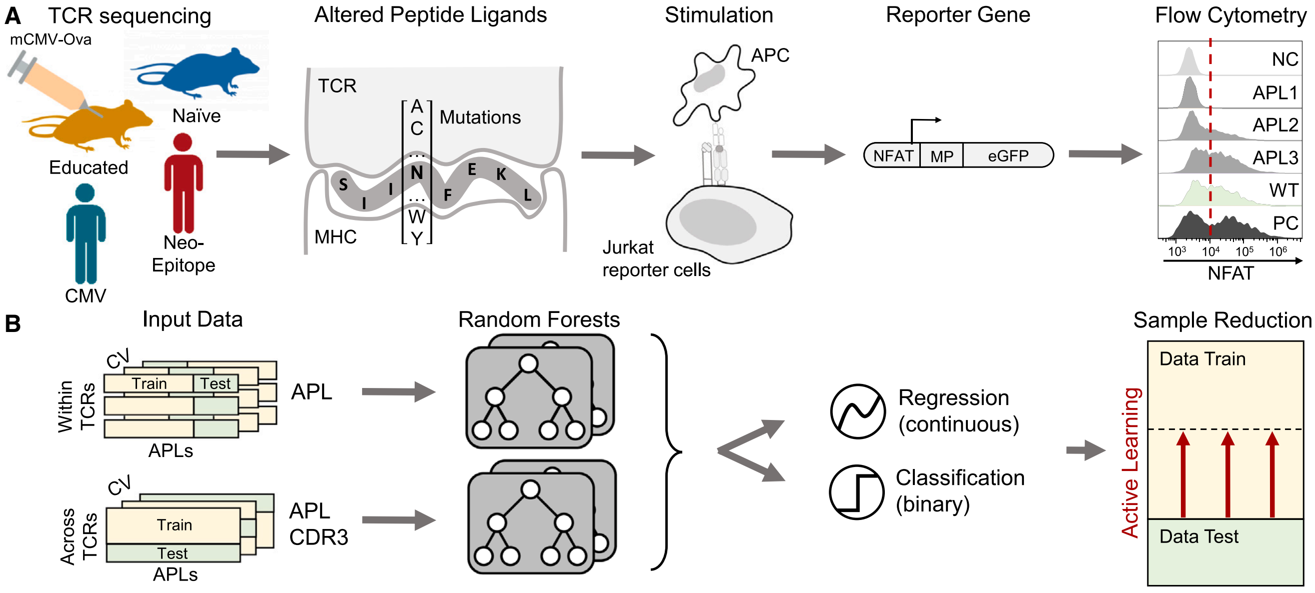

Figure 1. Overview of P-TEAM for predicting T cell activation by mutational epitopes

(A) Data acquisition: SIINFEKL-reactive TCRs were isolated based on H-2K^b-SIINFEKL multimers from mice previously exposed or unexposed to a murine CMV strain expressing SIINFEKL (mCMV-Ova). Two additional datasets consist of 6 previously identified human TCRs reactive to the tumor epitope VPSVWRSSL and 20 TCRs reactive to the CMV antigen NLVPMVATV. JTPR cells expressing a single TCR each were stimulated with APLs derived from single-amino acid mutations of the cognate epitopes. An activation score of each TCR toward each APL, a negative (NC) and positive control (PC), and the WT epitope is determined based on nuclear factor of activated T cells expression measured by flow cytometry after stimulation.

(B) Random forests predict the continuous activation score as a regression task, or the binary TCR recognition related to *in vivo* recruitment as a classification task, for all APLs within a TCR, as well as for an unseen TCR. The amount of training data can be reduced by efficient sampling using active learning techniques.

by measuring the effect of all single-point mutations of the well-characterized murine epitope SIINFEKL on TCR functional reactivity, and simultaneously determining T cell reactivity levels that correspond to actual recruitment and clonal expansion *in vivo* after pathogen infection (Figure 1A). In this work, we leverage this dataset to introduce Predicting T Cell Epitope-Specific Activation against Mutant Versions (P-TEAM), a random forest model trained to predict how T cell reactivity is affected by single-amino acid altered peptide ligands (APLs; Figure 1B).

The model can either learn from a TCR's reactivities toward a subset of APLs to predict the effect of the remaining mutations or generalize across a fully characterized TCR repertoire to novel TCRs. P-TEAM does not only classify TCR-epitope pairs as binding or non-binding but is also able to estimate a continuous activation score reflective of TCR reactivity. Additionally, we embedded P-TEAM into an active learning framework for experimental design to reduce the amount of training data required to obtain reliable predictions for novel epitopes. Finally, we applied P-TEAM on two datasets derived from human TCRs, revealing high performance in predicting potential cross-reactive epitopes for T cell-based immunotherapies.

RESULTS

Comprehensive quantification of T cell reactivity toward single-point mutations

To develop a model for predicting T cell functionality for mutational epitopes, we utilized the dataset described in Straub et al.,[14] which contains the functional reactivity information of 36 murine TCR sequences toward all single-mutation-based APLs of the model epitope SIINFEKL (murine dataset). The TCRs were either isolated from the naive repertoire of SIINFEKL-binding T cells (naive repertoire, n = 20) of an unexposed mouse[14] or the memory repertoire (educated repertoire, n = 15) of murine cytomegalovirus (mCMV)-SIINFEKL-exposed mice.[15] The dataset also included the well-studied SIINFEKL-reactive TCR OT-I as a reference control (n=1).

For each TCR, an activation score representing the fraction of activated T cells (Figure S1A) was experimentally determined for the wild-type (WT) epitope as well as all of its 152 APLs (8 positions × 19 amino acids = 152 APLs; STAR Methods, method details, data collection). In total, we studied 5,472 (36 TCRs × 152 APLs) unique murine TCR-peptide MHC (pMHC) interactions. The scores were normalized to allow comparisons across the different TCRs (Figure 2A). A TCR was marked as reactive to a peptide when the activation score exceeded 46.9%, which was identified as a threshold for effective recruitment and clonal expansion *in vivo*.[14]

Based on these experiments, the TCRs showed a broad range of functional reactivity levels (Figures 2B and S1B). Even though the TCRs were identified through binding toward H-2K^b/SIINFEKL multimers, 11 TCRs from the naive repertoire did not show any relevant reactivity toward the WT epitope or any APL upon transgenic re-expression in Jurkat triple parameter reporter (JTPR) cells, which were removed from all further experiments. Overall, the change in activation score is highly

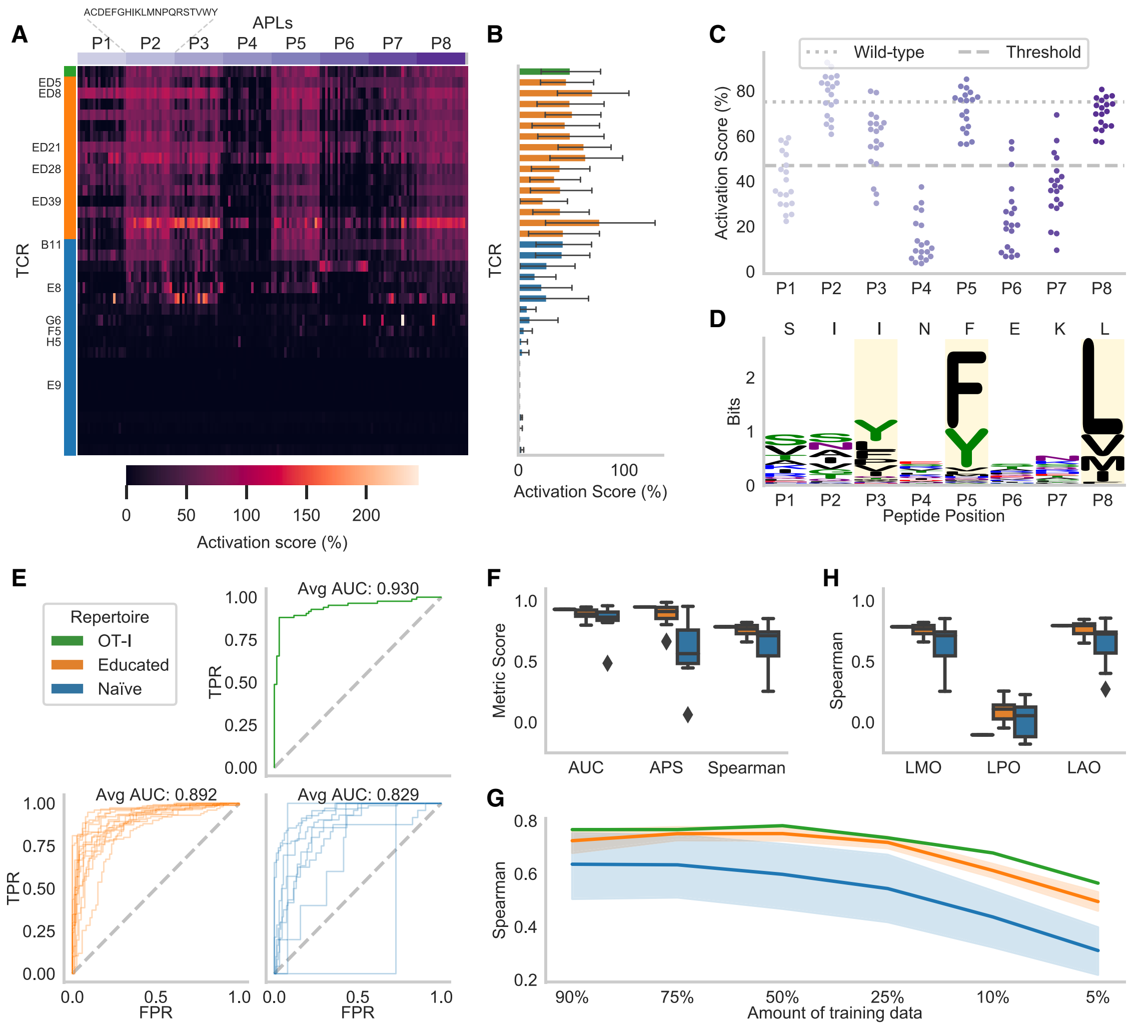

Figure 2. Predicting APL mutations within the reactivity landscape of a given TCR

(A) The normalized activation scores show a large variety of murine T cell activation in the deep mutational scan, in which each of the eight epitope positions (P1–P8) was exchanged by the other 19 amino acids in turn.

(B) The activation scores averaged for all APLs and the WT epitope (n = 153) of one TCR indicate high- and low-affinity TCRs.

(C) The epitope position on which the mutation occurs strongly influences the activation per APL (n = 19) averaged over all TCRs (n = 36). The threshold value represents the boundary between binding and non-binding, and WT indicates the activation scores of the base epitope SIINFEKL.

(D) MHC restrictiveness indicated by information content in bits for H-2K^b obtained from the MHC Motif Atlas[16] per position determined from n = 992 peptides. Reported anchor positions are highlighted in yellow and the WT epitope is indicated above.

(E) The receiver operating characteristic (ROC) curves for the different TCR repertoires indicate the TPR against the FPR at all prediction values as thresholds.

(F) Different evaluation metrics for regression (Spearman) and classification models.

(G) Spearman correlation when a smaller amount of training data is used (average over 10 repetitions with random subsets for each TCR).

(H) Spearman correlation obtained when trained on different subsets of the data.

The performance in (E)–(G) is shown for OT-I (n = 1 TCR), the educated repertoire (n = 15 TCRs), and the naive repertoire (n = 9 TCRs).

See also Figures S1–S6, Table S1, and STAR Methods, quantification and statistical analysis.

dependent on the position of the mutation (Figures 2C and S1C). Contrary to expectations, the TCR activation was not decreased by mutations at the H-2K^b anchor positions P3, P5, and P8, indicating that sufficient MHC binding might be retained by the remaining positions (Figures 2C and 2D). Rather, TCR activation is sensitive to mutations at the epitope positions P1, P4, P6, and P7, where H-2K^b allows a wide variety of amino acids. This was indicated by a strong Pearson correlation of 0.712 (p = 0.047) between the average activation score per position and the information content of the MHC motif positions,[16] which serves as a measure of how restrictive a position is (Figure S2A). Notably, these epitope positions were estimated to be in close proximity to the CDR3 of the TCR (Figure S2B) and probably contribute strongly to the TCR-epitope interaction. Therefore, mutations at the epitope center positions with low restrictiveness in their

MHC motif had, on average, a strong negative effect on T cell activation.

In summary, our dataset contains murine TCRs derived from the educated and the naive repertoires, with experimentally determined reactivities against all possible APLs. The TCRs from the educated repertoire showed reactivity against SIINFEKL and large numbers of its APLs, whereas TCRs from the naive repertoire showed overall fewer and more variable reactivities against the WT and mutant epitope versions.

P-TEAM predicts the effect of epitope point mutations on individual TCRs

We applied P-TEAM to this dataset to predict the effect of the various mutations of the epitope SIINFEKL for each TCR individually. Our approach is based on a random forest estimator, which receives physiochemical representations of amino acids[17] describing the WT epitope sequence and the APL sequences.

We first investigated whether it is possible to predict recognition of APLs by TCRs using a binary classification model. The random forest was trained separately for each TCR on 151 out of 152 APLs and predicted the probability of activation of the left-out mutation repeating this process for each APL. To evaluate the classification models, we reported the area under the receiver operating characteristic curve (AUC), which summarizes the true positive rate (TPR; recall, fraction of correct positive predictions over positive samples) and the false positive rate (FPR; incorrect positive predictions over negative samples) at all possible classification thresholds. We further provide the average precision score (APS), which indicates the AUC between precision (fraction of positive samples among those predicted as positive) and recall (see TPR). The performance (Table S1) on the educated repertoire was consistently high, with a mean AUC of 0.892 and APS of 0.883 among all TCRs (Figures 2E, 2F and S3). Only two receptors in the educated repertoire had an APS below 0.80—TCR ED28 (0.668) and TCR ED39 (0.662)—which showed the weakest cross-reactivity profile. The model for the reference TCR OT-I showed a similarly high performance as the educated repertoire (AUC: 0.930, APS: 0.949). The predictive performance decreased for the naive repertoire with a median AUC of 0.829 and APS of 0.581. This was expected given the more variable and overall lower reactivities against the WT epitope and APLs in this repertoire compared to the educated repertoire (Figures 2A and 2B). Consistent with this, the naive repertoire showed higher prediction variability between the different TCRs, reaching an AUC of 0.959 for TCR B11. The worst-performing TCRs achieved an APS of only 0.062 for TCR G6, which expressed reactivity to only five APLs, indicating difficulties in selecting recognized APLs when they occur rarely for a given TCR. However, the second-worst TCR, E8, followed after a great leap in performance of 0.447 in APS (Figures 2E, 2F, and S3), indicating that the TCRs of the naive repertoire were in fact predictable when they were reactive to a greater number of APLs. To evaluate the model by the position of mutation, we calculated the accuracy (fraction of correct predictions) as the AUC is ill-defined if all APLs at a position are assigned either nonreactive or reactive. On average, the model achieved a high accuracy of 0.866, showing a clear increase of 0.173 compared to classifying all APLs as either activating or not activating depending on which label occurred more frequently for a given TCR. While still outperforming this majority class prediction, the position-wise accuracy of P-TEAM decreased by 0.104 at epitope position P3 and by 0.054 at P7 (Figure S4), which were particularly difficult to predict as the average activation scores were close to the binarization threshold (Figure 2C).

Moving beyond binary classification, we also predicted the continuous reactivity of T cells in a regression setting for an APL in the same leave-mutation-out (LMO) validation scheme separated by TCR (Figure 2F) and evaluated the performance through Spearman's rank correlation (Table S1). The regression models showed similar variability as the classification models. However, the gap in median performance between the educated and naive repertoires was considerably greater: 13.8 percentage points for Spearman compared to 6.3 percentage points for AUC (Figure 2F). Furthermore, we evaluated whether models trained on binary data inherently learn continuous binding properties (Figure S5A)—in other words, whether the probability of reactivity predicted by the classification model also correlates with the actually measured reactivities. Intriguingly, the binding probability predicted by the classification model correlated to a large degree with the activation score for highly activated TCRs leading to a drop in the Spearman coefficient of only 0.050 for OT-I (Figure S5B) and 0.044 in the educated repertoire (Figure S5C).

Based on these results, we conclude that the TCR-epitope interaction can be predicted as a fine-grained continuous reactivity landscape beyond a binary recognition event.

Only 25% of random mutations are needed to learn a general model

We showed that P-TEAM can predict the T cell reactivity levels of a single mutation when trained on the remaining APLs. However, experimentally determining the activation of TCRs for the majority of, if not all, possible APLs comes with extensive labor, time, and cost expenses. Therefore, we analyzed the minimum number of APLs needed for training to obtain good generalization performance by comparing the performance of models trained on various subsets of APLs (Figures 2G, 2H, S6A, and S6B).

A given percentage of all APLs was randomly selected as training data, while the remaining samples were used for testing (Figures 2G and S6A). In the educated repertoire, the average performance decreased only slightly by 0.041 in the Spearman correlation and 0.026 in AUC when the model was trained on 25% of the available samples ($n = 38$ APLs) as compared to using all 151 APLs. When further reducing the training samples to 10% ($n = 15$ APLs), a noticeable drop in performance was observed, resulting in a decrease in the Spearman correlation of 0.147 and of 0.087 in AUC. The naive repertoire followed a similar pattern, albeit with decreased initial performance. The performance of the model remained stable until trained on only 25% of APLs ($n = 38$), however, with a generally larger decrease in performance (Spearman correlation: 0.078, AUC: 0.051) compared to the educated repertoire. This was followed by an even stronger decrease in performance (Spearman correlation: 0.186, AUC: 0.137) when trained on fewer data ($n = 15$ APLs).

To gain insights into the interaction between epitope features and predictions, we further evaluated the model in two

cross-validation settings by splitting the data either by amino acid or by position. In the first setting (leave-amino-acid-out [LAO]), all APLs containing a given amino acid, in turn, were reserved for validation, while in the second setting (leave-position-out [LPO]) the process was repeated based on epitope position instead of amino acids (Figures 2H and S6B). In the LAO setting, the regression performance changed only negligibly for both educated (Spearman: 0.015) and naive repertoires (Spearman: −0.017), suggesting that the model could successfully leverage the physiochemical features used to encode amino acids. However, when mutations at specific positions were left out in the training set, the model was barely able to predict T cell activation scores, resulting in a Spearman coefficient of 0.057 and an AUC of 0.495 across both repertoires, indicating random predictions. This highlights the importance of sampling across all epitope positions when predicting the mutational effects, and can be explained by the functional role of the different epitope positions.[18] While anchor positions fix the epitope within the MHC binding groove, and are therefore not accessible to the TCR, other positions are presented to the TCR to varying degrees and form the majority of interactions.

Accurate prediction of the reactivity landscape for unseen TCRs

In the previous experiments, the model predicted the effect of mutations on an individual TCR for which several APLs were observed during training. As a next step, we further evaluated the capability of our model to generalize to new TCRs by predicting the effect of all APLs on an unseen TCR that was held out during training (leave-TCR-out). In addition to the WT epitope and APL sequence, here, we provided the model with the sequence representation of the TCR CDR3α and CDR3β encoded by the Atchley factors, as described above.[17] Overall, this provided the model with a residue-level representation of the CDR3 regions from which common sequence features can be learned to generalize to related TCRs.

During classification for unseen TCRs, the AUC for the educated repertoire was 0.905 $\pm$ 0.041, and varied between 0.965 (TCR ED8) and 0.811 (TCR ED5) (Figures 3A, 3B, and S7; Table S1). The performance for the naive repertoire was considerably more variable than for the educated repertoire, with an average AUC of 0.620 $\pm$ 0.286 during classification. The three receptors with the smallest number of activated APLs had AUC values below 0.5, while the AUC for TCR OT-I was 0.959. This discrepancy between the predictive performance of different TCRs is not surprising. While receptors with high reactivity from the educated and naive repertoire interact with the APLs derived from SIINFEKL in a similar manner and are thereby predictable, low-reactive TCRs are likely to recognize different cognate epitopes, and hence follow widely different interaction patterns.[19] Overall, the classification of the model indicated by the AUC shows a strong statistically significant Pearson correlation of 0.911 to the WT activation (p =2.4 $\times$ 10^{-10}; Figure S8). Hence, we conclude that due to the composition of the dataset, our model is particularly suited for TCRs that show a high affinity toward the WT epitope.

To investigate the diversity in reaction patterns in the naive repertoire, we trained the classification model on all TCRs in the educated repertoire and predicted the activation scores of the naive repertoire (leave-naive-out) and vice versa (leave-educated-out). The classification performance measured by AUC scores obtained from the leave-educated-out model showed a large decrease in performance, with a mean absolute difference of 0.090 over the leave-TCR-out validation (Figure S9A). In contrast, the leave-naive-out method only led to a negligible maximal absolute difference of 0.025 (Figure S9B), providing additional evidence that the prediction was mainly driven by the educated repertoire, and TCRs in the naive repertoire were so diverse among one another that interaction patterns were not easily transferable from one TCR to the other.

To evaluate this further, we quantified the distance of each TCR toward the dataset by its mean TCRdist3[20] to the remaining TCRs. The classification performance of the model for both repertoires indicated by the AUC showed a statistically significant negative Spearman correlation ($\rho = -0.480$, $p = 0.015$; Figure 3C) to the TCR distance, indicating lower performance for less-related TCRs. This analysis strengthens the evidence that P-TEAM performs well on reasonably similar TCRs, and its performance is reduced to the degree to which the TCRs are different. These results indicate the possibility of further improving the performances of the model by acquiring experimental data covering a broader spectrum of binding modes.

P-TEAM outperforms conventional TCR-epitope binding predictors and epitope similarity measures

Recently, several machine learning approaches revealed good performance in classifying general pairs of TCRs and epitopes as binding or non-binding based on their sequences.[5–10] These tools are trained on large curated databases of publicly available TCR-epitope pairs, which only contain a limited amount of epitope mutations. Here, we report the comparative performance of the two deep learning-based predictors ImRex[10] and ERGO-II,[8] which were trained on general TCR epitope pairs, against the classification prediction of P-TEAM trained with the leave-TCR-out protocol on our mutation datasets to test whether specialized datasets are required to predict the effect of mutations (Figure 3D).

P-TEAM significantly outperformed all tested models (paired two-sided t test, all p < 0.0001) by a large margin (increase in averaged AUC over all TCRs larger than 0.25). This confirms the previous observations that these models often have decreased performance for unseen epitopes.[21] Overall, most baseline models performed only slightly better than random on this challenging dataset (average AUC < 0.60). However, this was expected as both predictors have, in contrast to P-TEAM, not encountered deep mutational scans, where small changes in the epitope sequences may cause large changes in activation patterns.

Intuitively, one could ask whether P-TEAM "simply" learns epitope similarities, compared to TCR-epitope interactions. To investigate this, we derived the WT epitope to APL similarities from the BLOSUM62 matrix,[22] indicating the likelihood of an amino acid exchange, and the Atchley factors,[17] which provide physiochemical summaries for the amino acids. As both similarity metrics neglect the position-dependent effect of a mutation, we set their binarization threshold per position to the value,

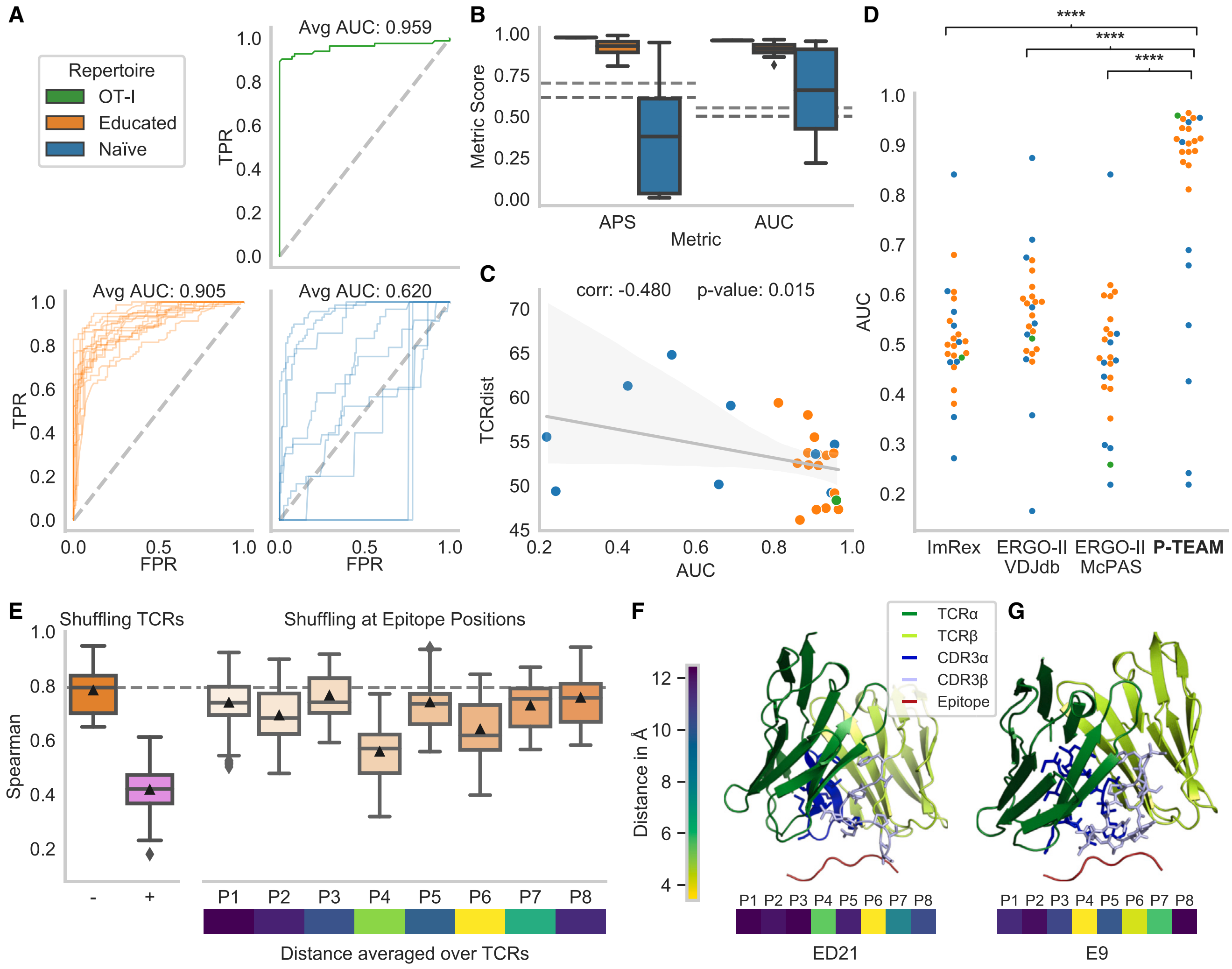

Figure 3. Generalization capabilities predicting activation for novel TCRs
(A) ROC curves for the different groups of TCRs.
(B) APS and AUC as additional classification metrics. The dashed line indicates the prediction using the labels of a random other TCR of the whole dataset (dark) or from the educated repertoire (light).
(C) Classification performance shows negative correlation to the average TCRdist3[20] between the training and test sets.
(D) P-TEAM significantly (****$p < 0.0001$) outperforms existing TCR-epitope predictors ImRex[10] and ERGO-II.[8]
The performance in (A)–(D) is shown for OT-I ($n = 1$ TCR), the educated repertoire ($n = 15$ TCRs), and the naive repertoire ($n = 9$ TCRs).
(E) The importance of input features obtained by replacing the test TCR input with a random CDR3 sequence of the dataset (+) or by shuffling the amino acid at each epitope position in the test set compared to the unshuffled performance (– and dashed line). The performance is indicated for all TCRs of the educated repertoire over repeated shuffling ($n = 15 \times 15 = 215$). Below, the average distance of the center of mass between the epitope and TCR residues is shown ($n = 32$ TCRs).
(F and G) Predicted structural model of the TCR and epitope, and minimal distance to the individual epitope positions for receptors ED21 and E9 (highest and lowest activation, respectively). The model shows the interaction between the epitope and the CDR3 of the TCRs.
See also Figures S7–S13, Table S1, and STAR Methods, quantification and statistical analysis.

resulting in the highest accuracy across the dataset and compared it to P-TEAM at the position-agnostic, unoptimized threshold of 50% classification probability. Despite this, P-TEAM outperformed BLOSUM62 and Atchley similarities on average by 0.128 and 0.139 in accuracy (Figure S10). Due to the optimized thresholds, epitope similarities performed on par with P-TEAM for positions where most APLs were either bound or not bound. However, they failed on the more variable positions P1, P3, and P7 in the educated repertoire.

These results highlight that current general TCR-epitope classifiers and epitope similarity measures cannot be used to predict T cell activation by APLs. Until the number of diverse epitopes and mutational scans in public databases increases drastically, specialized datasets and predictors such as P-TEAM are

Algorithm 1. Sample selection of P-TEAM

Data: S_{full}, S_{init}, A_{init}
$N_{add} \leftarrow 8$
$M \leftarrow 10$
$S_{train} \leftarrow S_{init}$
$A_{train} \leftarrow A_{init}$
for $i \leftarrow 1$ **to** M **do**
  classifier ← train_classifier(S_{train}, A_{init})
  uncertainty ← classifier($S_{full} \setminus S_{train}$)
  $S_{new} \leftarrow \varnothing$
  for $j \leftarrow 1$ **to** N_{add} **do**.
    $S_{new} \leftarrow S_{new}$ + argmax(uncertainty)
  end
  A_{new} ← experimentally_test(S_{new})
  $S_{train} \leftarrow S_{train} + S_{new}$
  $A_{train} \leftarrow A_{train} + A_{new}$
end

therefore needed to study the effect epitope mutations have on T cell activation.

P-TEAM learns biologically relevant interactions

To shed light on the inner workings of our model, we investigated the relevance of different input features in predicting TCR reactivity for unseen TCRs in a regression setting. We employed permutation importance tests to measure the contribution of features in the fitted model.[23] In short, the model is trained on the regular dataset; however, during the prediction phase, a set of input features is randomly perturbed. A large performance drop during this evaluation indicates that the model strongly relies on this information and is unable to perform accurate predictions if neglected. Here, we tested the importance of the full CDR3 region as well as each amino acid position individually (Figure 3E).

P-TEAM assigned the greatest importance to the CDR3 region, indicated by a drop of 0.366 in Spearman coefficient in the educated repertoire (Figure 3E). This behavior was expected as the TCRs show different activation patterns toward the epitope mutations. Hence, the model must incorporate the TCR sequence to generalize to unseen TCRs and not simply predict the activation score of a random, observed TCR. When analyzing epitope positions of SIINFEKL, the highest sensitivity was assigned to positions P4 and P6, with an absolute decrease in Spearman of 0.226 and 0.143, respectively (Figure 3E). In contrast, epitope positions P1, P3, P5, and P8 remained robust (decrease in Spearman <0.05), indicating the low importance of the residues at these positions for TCR binding. In fact, the side chains at these positions of SIINFEKL have previously been reported as completely (P5, P8) or predominantly (P1, P3) buried within the binding groove of MHC class I H-2K^b and, hence, are not in contact with the TCR.[24] The only exception is P2, which was also reported to be enclosed by the MHC but showed higher feature importance. Overall, this indicates that these positions do not have a strong impact on TCR-epitope binding and hardly influence prediction.

To further confirm that these results are in concordance with biological findings, we modeled the three-dimensional structure of each TCR and the epitope SIINFEKL using TCR-pMHC models.[25] The sensitive positions P4 and P6, which were previously identified to protrude from the binding groove,[26] laid in close proximity to the TCR, with a distance of less than 6 Å between the residues' center of mass when averaging across the murine dataset (Figures 3E–3G, S11, and S12), which corresponds to a distance that is indicative of contact between these residues.[27] Overall, the proximity of the epitope residues revealed a significantly strong correlation, with the feature importance indicated by a decrease in Spearman correlation (Pearson: 0.722, $p = 0.043$; Figure S13).

Overall, the feature importance analysis of our model, combined with findings in the literature and our structural modeling, further validated the results obtained by P-TEAM. The reliance of the model on known biologically relevant features ensures that its performance is not a statistical artifact but based on learning the interaction between APLs and TCRs.

Iterative experimental design decreases training set size to 24 APLs

As shown above, P-TEAM could accurately predict T cell activation while being trained with as few as 25% randomly selected APLs ($n = 38$) in a classification and regression setting. To further reduce the experimental effort required to train P-TEAM on new TCR repertoires, we optimized the experimental design to find the smallest subset of APLs needed to learn a well-performing model, as opposed to the random selection of APLs tested above. Active learning[28,29] is a collection of machine learning techniques that aim to iteratively improve the performance of a model, by deciding which samples to label experimentally (see Algorithm 1). These techniques require a small initial training dataset and further on request the label of additional examples, which are likely to improve the performance of the model the most by only collecting diverse and informative examples for training. In practice, this procedure requires multiple experiments to be performed sequentially in the wet lab with APLs suggested by the P-TEAM active learning framework. However, the total number of required samples, and therefore the total cost of collecting the dataset, is lowered through this alternating interplay between the acquisition of experimental data and model training.

We simulated this process by hiding the label for most APLs and gradually revealing the labels of a batch of examples ($n = 8$) whose prediction was most uncertain for the TCR-specific models (STAR Methods, active learning). We compared our active learning method with a baseline that randomly chooses eight APLs to label in each iteration. To start the active learning procedure, we provided an initial training dataset consisting of one APL per position with the amino acid exchange that was the most different from the WT epitope as quantified by the BLOSUM62 substitution probability.[22] Compared to a random selection, using this initialization set ($n = 8$) improved the performance noticeably in the regression task by an increase in Spearman correlation of 0.120 in the educated repertoire (Figure 4A), reaching an average Spearman correlation of 0.614 from only 8 observed samples. However, this is only reflected in a minor improvement during classification, with an absolute increase in AUC of 0.024 (Figure 4B) at the first iteration. At

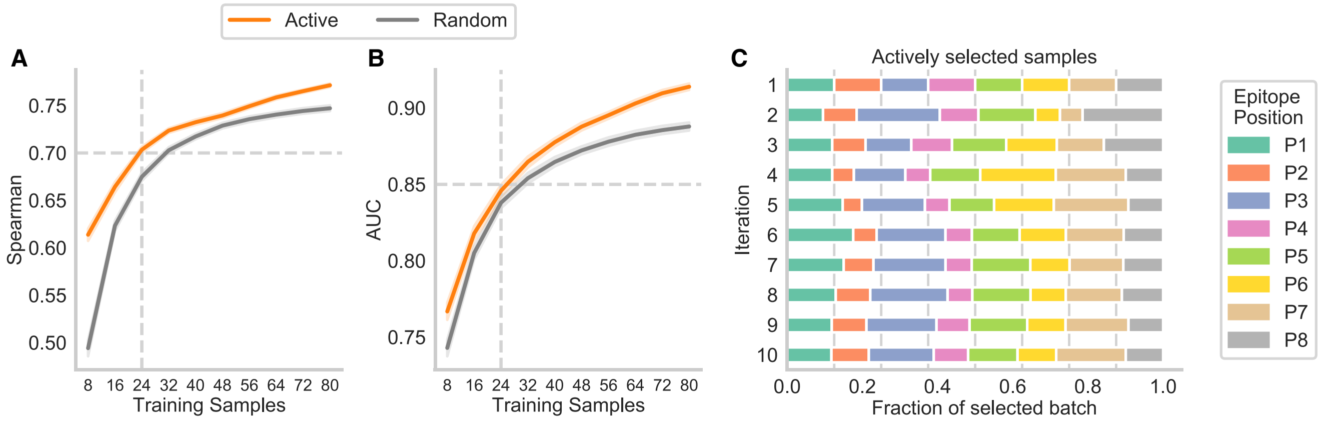

Figure 4. Reduction in training samples through active learning

(A and B) Comparison of the active learning framework to random sample selection on the educated repertoire of the murine dataset for classification (A) and regression models (B) for predicting within a TCR (n = 15 TCRs × 100 repetitions). The expected performance is shown for up to m = 10 consecutive iterations (N_{APLs} = 80) of alternating wet lab experiments and model training. The dashed horizontal line indicates the performance threshold of 0.7 Spearman and 0.85, respectively, which can be obtained by using three iterations (24 APLs) of active learning, as indicated by the dashed vertical line.

(C) Fraction of the mutated positions of the APLs within the newly selected training batch during the active learning process for each iteration. The vertical lines represent a random selection of the samples.

See also STAR Methods, quantification and statistical analysis.

iteration 10 (80 training APLs), the model achieves an AUC of 0.914, which required a training set of 137 random APLs in previous experiments (Figure S6A), thus reducing the amount of required training data by 42%. Overall, the active learning strategy statistically outperformed random sampling at every iteration on both datasets (unpaired t test, $p < 0.0001$ for regression and classification, with the exception of AUC at iteration 3, $p = 0.004$).

This improvement over random sampling can be attributed to a dataset-specific focus on certain positions. P-TEAM showed high uncertainty for mutations at positions P3 and P8 at the second iteration, leading to an oversampling with 22.1% and 21.4%, respectively, of the selected APLs stemming from these positions (Figure 4C). Over all 10 iterations, APLs with mutations at position P3 were sampled most frequently (17.1% of the training samples). Conversely, positions P2 and P4 were selected the least, with a frequency of 8.4% and 8.5%, respectively, as T cell reactivity was comparable for all exchanges at these positions (Figures 2A and 2C). With this interplay between experimental design and computational modeling, P-TEAM was able to reach a high performance of AUC > 0.85 and Spearman > 0.70 after the third iteration. We, therefore, conclude that three experimental rounds of alternating wet-lab and *in silico* experiments, collecting 24 APLs of an 8mer epitope (15.8% of the dataset) in total, are sufficient to train P-TEAM to a satisfactory performance level.

P-TEAM identifies cross-reactive APLs for neo-epitope-specific human TCRs

To further validate P-TEAM on a therapeutically relevant epitope, we introduced a second mutational scan for the human cancer neo-epitope VPSVWRSSL (STAR Methods, data collection; Figures S14A–S14C). The human leukocyte antigen (HLA)-B*07:02-restricted epitope VPSVWRSSL occurs in a frame-shift-induced neo-open reading frame of the gene RNF43,[30,31] which is frequently mutated in gastrointestinal cancers.[32] Seven HLA-B*07:02/VPSVWRSSL-binding TCRs with therapeutic potential were isolated from healthy donors, of which six TCRs showed reactivity against the WT epitope and several of the 133 tested APLs (798 unique human pMHC-TCR interactions). The dataset did not contain 38 APLs predicted to break MHC binding via NetMHCpan,[33] which occurred especially for mutations at anchor positions P2 and P9 affecting 19 and 15 APLs, respectively. Contrary to the murine dataset, all TCRs of the neo-epitope dataset recognized at least 20 mutations, with a maximum of 75 mutations (Figure 5A). Two TCRs, R24 and R28, showed the least overall activation (Figure 5B) caused by limited reactivity at the end positions P7–P9. Overall, the change in activation score was again highly dependent on the position of the mutation. Exchanges at center positions P4, P5, and P6 without MHC restrictions indicated in the HLA-B*07:02 motif generally led to a drop in activation (Figures 5C and 5D).

During LMO classification, P-TEAM performed similarly to the educated repertoire, with an average AUC of 0.890 and APS of 0.784 (Figures 5E and 5F; Table S2), with the worst-performing TCR R24 (AUC: 0.761, APS: 0.322) and the best-performing TCR R25 (AUC: 0.949, APS: 0.933). Predicting the activation score in the regression setting showed high performance, with a Spearman correlation of 0.734 (Figure 5F) in the neo-epitope dataset indicating that the approach can be applied to different epitopes as well as human TCRs.

As in the murine dataset, the model performed only slightly worse when trained on 25% of the neo-epitope APLs (n = 33; Figures S15A and S15B), with a decrease of 0.044 in Spearman correlation and a decrease of 0.024 in AUC. Again, active learning further improved sample efficiency for the neo-epitope dataset (Figures 5G and 5H). With the initial set, the model reached a Spearman correlation of 0.663 and an AUC of 0.810,

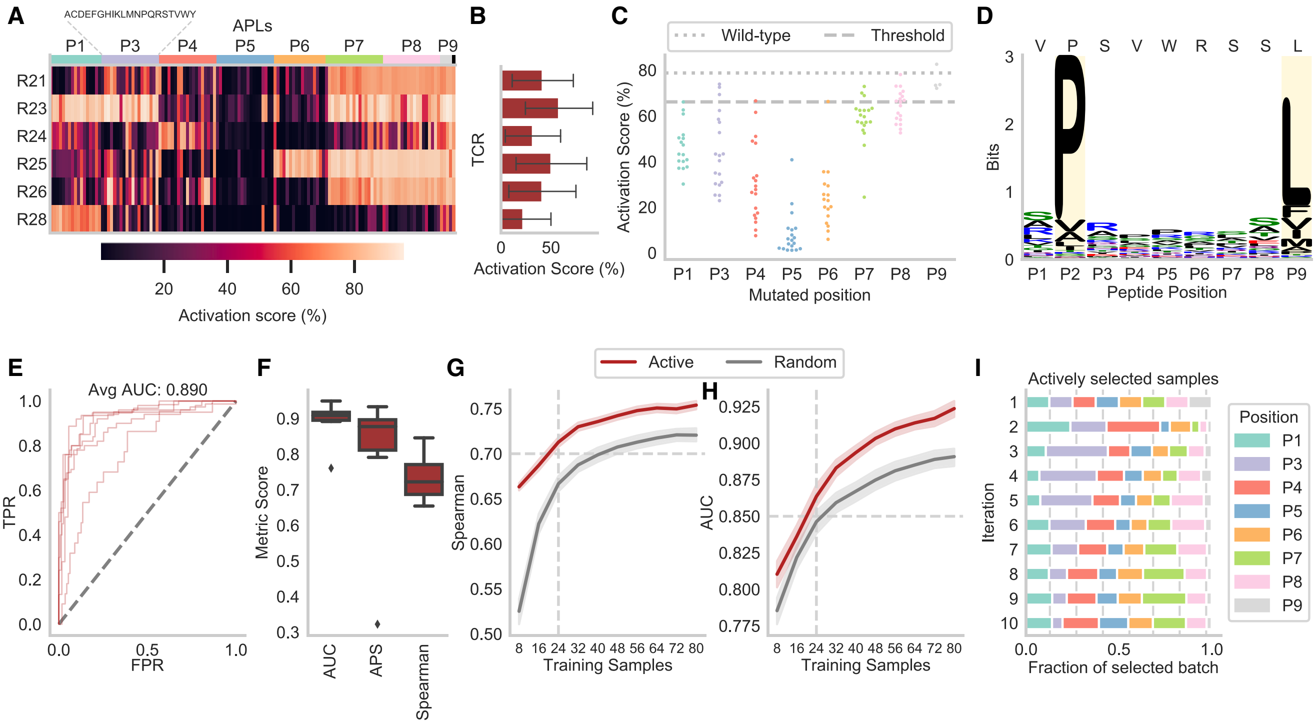

Figure 5. Predicting the effect of neo-epitope mutations within a human TCR

(A) The normalized activation scores of six TCRs of the neo-epitope dataset express high activation against the mutation landscape.

(B) The activation scores averaged for all APLs (n = 133) of one TCR indicate two reactivity patterns.

(C) The epitope position on which the mutation occurs strongly influences the activation per APL (n = 19, except P1: n = 17, P6: n = 17, and P9: n = 4) averaged over all TCRs (n = 6). The threshold value represents the boundary between binding and non-binding, and WT indicates the activation scores of the base epitope VPSVWRSSL.

(D) MHC restrictiveness indicated by information content in bits for HLA-B*07:02 obtained from n = 6,747 peptides in the MHC Motif Atlas[16] per position. Reported anchor positions are highlighted in yellow and the WT epitope is indicated above.

(E) The ROC curves of the six neo-epitope-specific TCRs indicate the TPR against the FPR at all prediction values as thresholds.

(F) Different evaluation metrics for regression (Spearman) and classification models (n = 6 TCRs).

(G and H) Comparison of the active learning framework to random sample selection on the neo-epitope dataset for regression (G) and classification models (H) (n = 6 TCRs × 100 repetitions). The expected performance is shown for up to m = 10 consecutive iterations (N_{APLs} = 80) of alternating wet lab experiments and model training. The dashed horizontal line indicates the performance threshold of 0.7 Spearman and 0.85, respectively, which can be obtained by using three iterations (24 APLs) of active learning as indicated by the dashed vertical line.

(I) Fraction of the mutation positions of the APLs within the newly selected training batch during the active learning process for each iteration. The vertical lines represent a random selection of the samples.

See also Figures S14 and S15, Table S2, and STAR Methods, quantification and statistical analysis.

outperforming random sampling by 0.138 and 0.025, respectively. In the following iterations, the model steadily improved to a Spearman correlation of 0.754 and an AUC of 0.924 at iteration 10 (n = 80 APLs), significantly outperforming random sample selection at every iteration (unpaired t test, $p < 0.0001$ for classification and regression, with the exception of AUC at iteration 1, $p = 0.015$). At the threshold of $n = 24$ APLs, P-TEAM was able to predict the T cell activation with an AUC of 0.864 and a Spearman correlation of 0.713. As in the murine dataset (Figure 4C), APLs with exchanges at position P3 were identified as beneficial to the training process, with a frequency of 19.5% at the first iteration and 18.1% in total (Figure 5I). However, contrary to the murine dataset, positions P1 and P4 were heavily oversampled at iteration 1, with 23.9% and 28.9% in the neo-epitope dataset. This focus on the differing positions between the two datasets emphasizes the need for an uncertainty-based experimental design, as no generalized rules can be derived across different epitopes.

Contrary to the LMO experiments, the average model performance was reduced compared to the educated repertoire (AUC: 0.771, APS: 0.663, Spearman: 0.663) for predictions in the leave-TCR-out setting (Figures 6A–6C; Table S2). Specifically, the model failed to generalize to the two TCRs, R24 and R28, with an AUC of 0.561 and 0.618, respectively, which followed different activation patterns at positions P7–P9 (Figure 5A). Presumably, a larger variety in binding modes needs to be captured within the repertoire data to further generalize to unseen TCRs with varying activation patterns. Despite the low performance on these two TCRs, the model outperformed the general TCR-epitope predictors by an increase in AUC of 0.116–0.289 (Figure 6D).

Feature importance was predominantly assigned to epitope position P5 (decrease in Spearman: 0.226; Figure 6E) that lay with an

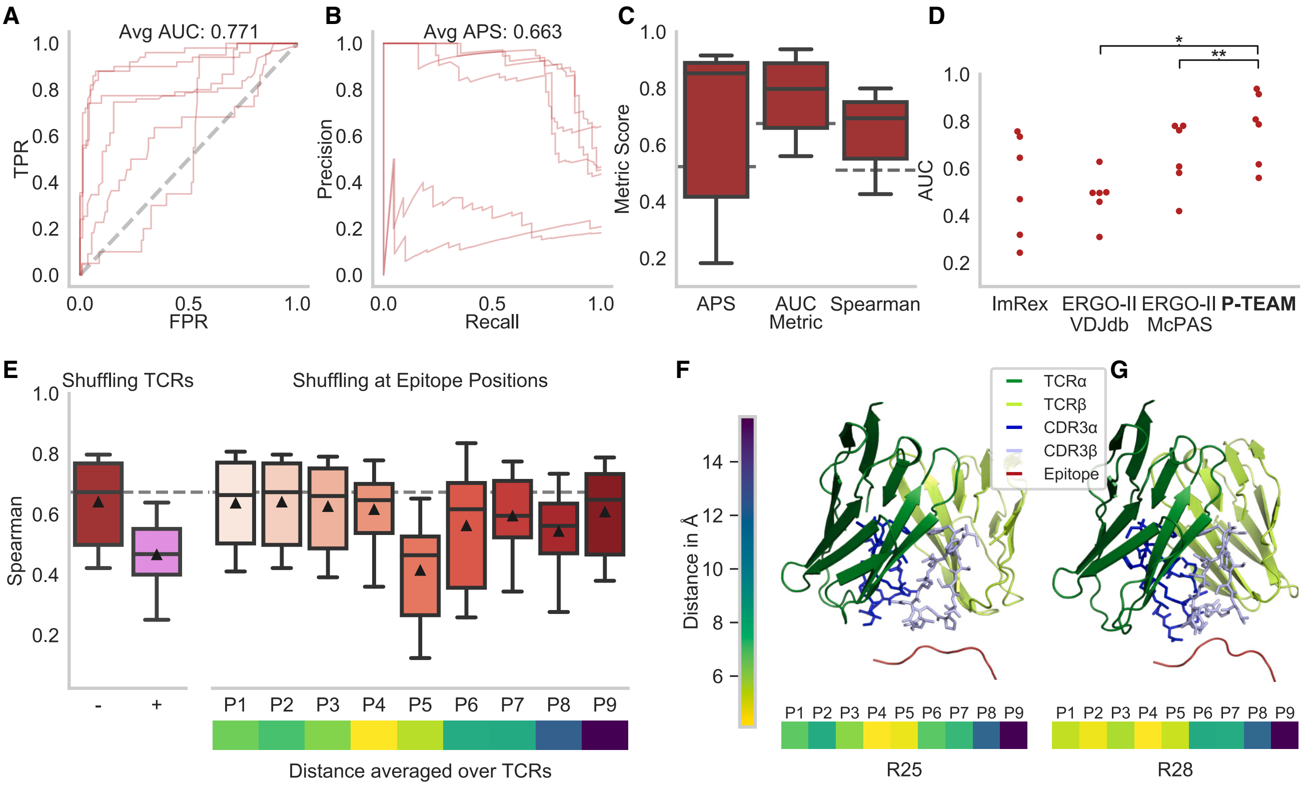

Figure 6. Across-repertoire prediction for the neo-epitope dataset

(A and B) ROC (A) and precision-recall (B) curves for the six TCRs of the neo-epitope dataset.

(C) APS, AUC, and Spearman correlation as classification and regression metrics. The dashed line indicates the prediction using the labels of a random other TCR.

(D) P-TEAM outperforms existing TCR-epitope predictors ImRex[10] and ERGO-II[8] by a large margin (*$p < 0.05$; **$p < 0.01$).

(A–E) Performance over $n = 6$ TCRs.

(E) The importance of input features obtained by replacing the test TCR input with a random CDR3 sequence of the dataset (+) or by shuffling the amino acid at each epitope position in the test set compared to the unshuffled performance (– and dashed line) ($n = 6$ TCRs × 15 repetitions). Below, the average distance of the center of mass between the epitope and TCR residues is shown ($n = 6$).

(F and G) Predicted structural model of the TCR and epitope, and minimal distance to the individual epitope positions for receptors R25 and R28 (highest and lowest activation, respectively). The model shows the interaction between the epitope and the CDR3 of the TCRs.

See also Figure S16, Table S2, and STAR Methods, quantification and statistical analysis.

average distance of 5.64 Å, second closest to the TCR in the structural models[25] (Figures 6F, 6G and S16). The effects of the anchor positions could not be observed as the dataset contained only four APLs with mutations at P9 and none for P2 since the remaining mutations were not experimentally determined. However, P3, which was ranked second to last in importance (decrease in Spearman: 0.024), had been reported to form an optional stabilizing interaction to the MHC class I HLA-B07*02.[34] The predicted spatial models show that the two outlier TCRs, R24 and R28, also follow different structural patterns. Both TCRs lay closer to positions P1 and P2 than the remaining TCRs (Figures 6F, 6G, and S16), which might further indicate a different TCR-pMHC interaction pattern. However, it must be noted here that structural interpretation on the neo-epitope dataset must be viewed with caution as the underlying template epitope in TCR-pMHC models[25] expressed only 33.3% sequence identity to VPSVWRSSL.

To summarize, P-TEAM achieved high performance in predicting the effects of mutations in the neo-epitope VPSVWRSSL on T cell reactivity during classification and regression, even though the leave-TCR-out setting is slightly limited due to the low amount of available TCRs. The model adapted to the changing effects of mutations at specific epitope positions as shown through changes in feature importance and differing sample selection during active learning.

Effects of mutations in viral epitopes are predictable

To showcase P-TEAM in other disease settings, we introduced a third deep mutational scan against the commonly studied human CMV epitope pp65 NLVPMVATV restricted to HLA-A*02:01 (STAR Methods, data collection). The scan comprised the activation of 20 TCRs against all 171 mutated APLs, resulting in 3,420 unique TCR-epitope interactions (Figure S17A). The TCRs were activated with a mixed landscape from 3 to 99 activating APLs (Figure S17B) resembling the reactivity spectra of the combined naive and educated murine dataset. The epitope positions P4 and P5 without MHC restrictions were highly

sensitive toward mutations in addition to the HLA-A*02:01 anchor position P9 (Figures S17C and S17D), where mutations, presumably, broke MHC binding. In contrast to the murine repertoire, we did not observe any correlation between MHC restrictiveness and the average activation score per position (Figure S17E).

On average, P-TEAM achieved an AUC of 0.728 and a Spearman correlation of 0.592 for predictions of individual TCRs (Figures S17F and S17G; Table S3), showing a considerably larger variance than in the murine datasets. The model mainly failed with an AUC <0.60 for 3 out of 20 TCRs for which only limited APLs were indicated as binding (Figure S17H). As for the two other datasets, the utilization of active learning improved the performance at every iteration, albeit on an overall reduced level (Figures S17I and S17J). Compared to LMO prediction, the performance of P-TEAM increased in the leave-TCR-out setting, reaching a Spearman correlation of 0.788 and an AUC of 0.884 (Figures S18A–S18C), thus significantly surpassing general TCR-epitope predictors by >0.30 (p <0.0001; Figure S18D; Table S3). Again, the largest importance was assigned to the CDR3 sequence (Figure S18E), followed by epitope position P1 and position P5, which was estimated to be on average closest to the TCRs (Figures S18F, S18G, and S19).

Based on the results of these two datasets, we therefore conclude that P-TEAM can be applied to therapeutically relevant TCRs across different host organisms, epitopes, MHC alleles, and diseases, if a sufficient number of annotated samples is available.

DISCUSSION

Pathogens and cancer cells try to escape surveillance by the adaptive immune system through epitope mutations that prevent TCR binding. Indeed, single-point mutations can be enough to evade previously formed immune memory.[35] While even general TCR binding prediction remains a challenge, predicting the effect of point mutations is especially difficult, as public datasets used as training data contain very few examples of epitopes differing by one residue toward the same receptor.

As a first step toward this goal, we introduced P-TEAM, a single-point mutational effect predictor trained on three datasets that measured TCR reactivity levels for single-point mutations of three different epitope-MHC combinations comprising a total of 9,690 TCR-APL unique interactions. We modeled the interaction of T cells to epitope mutation for individual TCRs, as well as across repertoires, with high accuracy, indicating the validity of our approach for different epitopes and host organisms. The model was able to learn this interaction based on the APL and TCR CDR3 sequences even when trained on a limited number of annotated samples. While most prediction methods treat the TCR-epitope interaction as a binary event of recognition or non-recognition, P-TEAM could predict not only such a classification but also a continuous reactivity score in a regression setting.

In general, T cell activation was sensitive to mutations at the epitope's center positions that were not restricted by the MHC. Thus, MHC motifs[16] can serve as an initial indicator for the effect of mutations but are agnostic to changing binding modes between TCRs leading to different activation patterns. We found that the predictions of the model were driven by these highly sensitive residues in the epitope, which differed between different WT epitopes. Based on the predicted spatial proximity of epitope and TCR residues, we validated that the model extracted meaningful interactions of the TCRs to residues of the APLs, which are in line with previous findings in the literature. TCR-specific models could be trained with one-quarter of all possible mutations (n = 38) without any notable changes in prediction performance. This amount was further reduced to 24 samples (15% of the APLs) by alternating experimental design framework between wet lab experiments and model training.

In conclusion, we present P-TEAM, a TCR-epitope binding predictor specializing in single-point mutations of epitopes that generalizes across receptors and can be trained with as few as 24 mutations. The model is able to estimate continuous activation that ultimately characterizes TCR-epitope interactions beyond binary recognition. Our findings point to the intriguing possibility of predicting changes in T cell functionality due to single-point mutations in a quantitative manner from epitope and TCR sequence alone. P-TEAM therefore bears the potential of improving the safety and effectiveness of immunotherapies and vaccines.

Limitations of the study

While we demonstrated P-TEAM on three diverse datasets, more epitope-MHC combinations would provide further validation of the broad applicability of the model. Furthermore, we focused on MHC class I epitopes in this study, while the predictability of mutations in MHC class II epitopes remains to be tested. Currently, our modeling approach incorporates only the APL and CDR3 sequences to guide the predictions. The advantages of including V(D)J-gene types, or pretrained TCR embeddings such as TCRbert,[36] remain untested, which could guide P-TEAM toward better generalized predictions through an improved TCR representation. While we reduce the amount of training data through active learning, P-TEAM is applicable only when the effect of several mutations on a TCR is known. However, recent advances in structural modeling such as AlphaFold3[37] and similar methods might be harnessed to accurately model the TCR-peptide-MHC structure. Such models could allow us to investigate the mechanisms in their interaction, such as pMHC-complex rigidity and advantageous TCR-peptide contacts,[2] from a holistic perspective of all three components at large scale, and thereby obtain a model that generalizes to novel epitopes.

STAR★METHODS

Detailed methods are provided in the online version of this paper and include the following:

- KEY RESOURCES TABLE
- RESOURCE AVAILABILITY
  - Lead contact
  - Materials availability
  - Data and code availability
- EXPERIMENTAL MODEL AND STUDY PARTICIPANT DETAILS
  - Cell lines
- METHOD DETAILS
  - CRISPR-Cas9-mediated orthotopic TCR replacement
  - Generation of retroviruses and transduction
  - Data collection

- Predictors
- Perturbation tests
- Baseline TCR predictors and distances
- Structural modeling
- Active learning
- QUANTIFICATION AND STATISTICAL ANALYSIS

SUPPLEMENTAL INFORMATION

Supplemental information can be found online at https://doi.org/10.1016/j.xgen.2024.100634.

ACKNOWLEDGMENTS

E.D. and F.D. are supported by the Helmholtz Association under the joint research school Munich School for Data Science – MUDS. F.D. acknowledges financial support from the Joachim Herz Stiftung. K.S. is supported by the BMBF (project 01KI2013). P.H. is supported by the Else Kröner-Stiftung (project 2020_EKEA.127). D.H.B. was supported by the Deutsche Forschungsgemeinschaft (DFG) SFB-TRR 338/1 2021 -452881907 (project A01). This work was supported mainly by BMBF grant DeepTCR (project 031L0290A) to K.S. and B.S.

AUTHOR CONTRIBUTIONS

B.S. and K.S. conceived the project. E.D. and F.D. performed the research, implemented the models, and performed the analysis. A.S., P.H., K.I.W., K.H., M.L.M., and D.H.B. acquired the experimental data. B.S., K.S., D.H.B., and B.B. supervised the research. All authors wrote the manuscript.

DECLARATION OF INTERESTS

The authors declare no competing interests.

Received: June 13, 2023
Revised: April 22, 2024
Accepted: July 22, 2024
Published: August 15, 2024

REFERENCES

1. Bentzen, A.K., Such, L., Jensen, K.K., Marquard, A.M., Jessen, L.E., Miller, N.J., Church, C.D., Lyngaa, R., Koelle, D.M., Becker, J.C., et al. (2018). T cell receptor fingerprinting enables in-depth characterization of the interactions governing recognition of peptide-mhc complexes. Nat. Biotechnol. *36*, 1191–1196. https://doi.org/10.1038/nbt.4303.
2. Bovay, A., Zoete, V., Rizkallah, P.J., Beck, K., Delbreil, P., Speiser, D.E., Cole, D.K., and Fuertes Marraco, S.A. (2020). Identification of a superagonist variant of the immunodominant yellow fever virus epitope ns4b 214-222 by combinatorial peptide library screening. Mol. Immunol. *125*, 43–50. https://doi.org/10.1016/j.molimm.2020.06.025.
3. Linette, G.P., Stadtmauer, E.A., Maus, M.V., Rapoport, A.P., Levine, B.L., Emery, L., Litzky, L., Bagg, A., Carreno, B.M., Cimino, P.J., et al. (2013). Cardiovascular toxicity and titin cross-reactivity of affinity-enhanced T cells in myeloma and melanoma. Blood *122*, 863–871. https://doi.org/10.1182/blood-2013-03-490565.
4. Zarnitsyna, V.I., Evavold, B.D., Schoettle, L.N., Blattman, J.N., and Antia, R. (2013). Estimating the diversity, completeness, and cross-reactivity of the t cell repertoire. Front. Immunol. *4*, 485. https://doi.org/10.3389/fimmu.2013.00485.
5. De Neuter, N., Bittremieux, W., Beirnaert, C., Cuypers, B., Mrzic, A., Moris, P., Suls, A., Van Tendeloo, V., Ogunjimi, B., Laukens, K., and Meysman, P. (2018). On the feasibility of mining cd8+ t cell receptor patterns underlying immunogenic peptide recognition. Immunogenetics *70*, 159–168. https://doi.org/10.1093/bib/bbaa318.
6. Gielis, S., Moris, P., Bittremieux, W., De Neuter, N., Ogunjimi, B., Laukens, K., and Meysman, P. (2019). Detection of enriched t cell epitope specificity in full t cell receptor sequence repertoires. Front. Immunol. *10*, 2820. https://doi.org/10.3389/fimmu.2019.02820.
7. Jokinen, E., Huuhtanen, J., Mustjoki, S., Heinonen, M., and Lähdesmäki, H. (2021). Predicting recognition between t cell receptors and epitopes with tcrgp. PLoS Comput. Biol. *17*, e1008814–e1008827. https://doi.org/10.1371/journal.pcbi.1008814.
8. Springer, I., Tickotsky, N., and Louzoun, Y. (2021). Contribution of t cell receptor alpha and beta cdr3, mhc typing, v and j genes to peptide binding prediction. Front. Immunol. *12*, 1436. https://doi.org/10.1023/A:1010933404324.
9. Weber, A., Born, J., and Rodriguez Martínez, M. (2021). Titan: T-cell receptor specificity prediction with bimodal attention networks. Bioinformatics *37*, i237–i244. https://doi.org/10.1093/bioinformatics/btab294.
10. Moris, P., De Pauw, J., Postovskaya, A., Gielis, S., De Neuter, N., Bittremieux, W., Ogunjimi, B., Laukens, K., and Meysman, P. (2021). Current challenges for unseen-epitope tcr interaction prediction and a new perspective derived from image classification. Brief. Bioinform. *22*, bbaa318. https://doi.org/10.1093/bib/bbaa318.
11. Vita, R., Mahajan, S., Overton, J.A., Dhanda, S.K., Martini, S., Cantrell, J.R., Wheeler, D.K., Sette, A., and Peters, B. (2019). The immune epitope database (IEDB): 2018 update. Nucleic Acids Res. *47*, D339–D343. https://doi.org/10.1093/nar/gky1006.
12. Bagaev, D.V., Vroomans, R.M.A., Samir, J., Stervbo, U., Rius, C., Dolton, G., Greenshields-Watson, A., Attaf, M., Egorov, E.S., Zvyagin, I.V., et al. (2020). VDJdb in 2019: database extension, new analysis infrastructure and a T-cell receptor motif compendium. Nucleic Acids Res. *48*, D1057–D1062. https://doi.org/10.1093/nar/gkz874.
13. Hwang, J.-R., Byeon, Y., Kim, D., and Park, S.-G. (2020). Recent insights of t cell receptor-mediated signaling pathways for t cell activation and development. Exp. Mol. Med. *52*, 750–761. https://doi.org/10.1038/s12276-020-0435-8.
14. Straub, A., Grassmann, S., Jarosch, S., Richter, L., Hilgendorf, P., Hammel, M., Wagner, K.I., Buchholz, V.R., Schober, K., and Busch, D.H. (2023). Recruitment of epitope-specific t cell clones with a low-affinity threshold supports efficacy against mutational escape upon re-infection. Immunity *56*, 1269–1284.e6. https://doi.org/10.1016/j.immuni.2023.04.010.
15. Schober, K., Voit, F., Grassmann, S., Müller, T.R., Eggert, J., Jarosch, S., Weißbrich, B., Hoffmann, P., Borkner, L., Nio, E., et al. (2020). Reverse tcr repertoire evolution toward dominant low-affinity clones during chronic cmv infection. Nat. Immunol. *21*, 434–441. https://doi.org/10.1038/s41590-020-0628-2.
16. Tadros, D.M., Eggenschwiler, S., Racle, J., and Gfeller, D. (2023). The mhc motif atlas: a database of mhc binding specificities and ligands. Nucleic Acids Res. *51*, D428–D437. https://doi.org/10.1093/nar/gkac965.
17. Atchley, W.R., Zhao, J., Fernandes, A.D., and Drüke, T. (2005). Solving the protein sequence metric problem. Proc. Natl. Acad. Sci. USA *102*, 6395–6400. https://doi.org/10.1073/pnas.0408677102.
18. Calis, J.J.A., De Boer, R.J., and Keşmir, C. (2012). Degenerate t-cell recognition of peptides on mhc molecules creates large holes in the t-cell repertoire. PLoS Comput. Biol. *8*, e1002412. https://doi.org/10.1371/journal.pcbi.1002412.
19. Szeto, C., Lobos, C.A., Nguyen, A.T., and Gras, S. (2020). Tcr recognition of peptide-mhc-i: rule makers and breakers. Int. J. Mol. Sci. *22*, 68. https://doi.org/10.3390/ijms22010068.
20. Mayer-Blackwell, K., Schattgen, S., Cohen-Lavi, L., Crawford, J.C., Souquette, A., Gaevert, J.A., Hertz, T., Thomas, P.G., Bradley, P., and Fiore-Gartland, A. (2021). Tcr meta-clonotypes for biomarker discovery with tcrdist3 enabled identification of public, hla-restricted clusters of sars-cov-2 tcrs. Elife *10*, e68605. https://doi.org/10.7554/eLife.68605.
21. Hudson, D., Fernandes, R.A., Basham, M., Ogg, G., and Koohy, H. (2023). Can we predict t cell specificity with digital biology and machine learning?

Nat. Rev. Immunol. *23*, 511–521. https://doi.org/10.1038/s41577-023-00835-3.

22. Henikoff, S., and Henikoff, J.G. (1992). Amino acid substitution matrices from protein blocks. Proc. Natl. Acad. Sci. USA *89*, 10915–10919. https://doi.org/10.1073/pnas.89.22.10915.
23. Breiman, L. (2001). Random forests. Mach. Learn. *45*, 5–32. https://doi.org/10.1023/A:1010933404324.
24. Fremont, D.H., Matsumura, M., Stura, E.A., Peterson, P.A., and Wilson, I.A. (1992). Crystal structures of two viral peptides in complex with murine mhc class i h-2kb. Science *257*, 919–927. https://doi.org/10.1126/science.1323877.
25. Jensen, K.K., Rantos, V., Jappe, E.C., Olsen, T.H., Jespersen, M.C., Jurtz, V., Jessen, L.E., Lanzarotti, E., Mahajan, S., Peters, B., et al. (2019). Tcrpmhcmodels: Structural modelling of tcr-pmhc class i complexes. Sci. Rep. *9*, 14530. https://doi.org/10.1038/s41598-019-50932-4.
26. Fremont, D.H., Stura, E.A., Matsumura, M., Peterson, P.A., and Wilson, I.A. (1995). Crystal structure of an h-2kb-ovalbumin peptide complex reveals the interplay of primary and secondary anchor positions in the major histocompatibility complex binding groove. Proc. Natl. Acad. Sci. USA *92*, 2479–2483. https://doi.org/10.1073/pnas.92.7.2479.
27. Fout, A., Byrd, J., Shariat, B., and Ben-Hur, A. (2017). Protein interface prediction using graph convolutional networks. Adv. Neural Inf. Process. Syst. *30*.
28. Ren, P., Xiao, Y., Chang, X., Huang, P.Y., Li, Z., Gupta, B.B., Chen, X., and Wang, X. (2021). A survey of deep active learning. ACM Comput. Surv. *54*, 1–40. https://doi.org/10.1145/3472291.
29. Mohamed, T.P., Carbonell, J.G., and Ganapathiraju, M.K. (2010). Active learning for human protein-protein interaction prediction. BMC Bioinf. *11*, S57–S59. https://doi.org/10.1186/1471-2105-11-S1-S57.
30. Amato, E., Molin, M.D., Mafficini, A., Yu, J., Malleo, G., Rusev, B., Fassan, M., Antonello, D., Sadakari, Y., Castelli, P., et al. (2014). Targeted next-generation sequencing of cancer genes dissects the molecular profiles of intraductal papillary neoplasms of the pancreas. J. Pathol. *233*, 217–227. https://doi.org/10.1002/path.4344.
31. Tu, J., Park, S., Yu, W., Zhang, S., Wu, L., Carmon, K., and Liu, Q.J. (2019). The most common RNF43 mutant G659Vfs*41 is fully functional in inhibiting Wnt signaling and unlikely to play a role in tumorigenesis. Sci. Rep. *9*, 18557. https://doi.org/10.1038/s41598-019-54931-3.
32. Loregger, A., Grandl, M., Mejías-Luque, R., Allgäuer, M., Degenhart, K., Haselmann, V., Oikonomou, C., Hatzis, P., Janssen, K.P., Nitsche, U., et al. (2015). The e3 ligase rnf43 inhibits wnt signaling downstream of mutated β-catenin by sequestering tcf4 to the nuclear membrane. Sci. Signal. *8*, ra90. https://doi.org/10.1126/scisignal.aac6757.
33. Reynisson, B., Alvarez, B., Paul, S., Peters, B., and Nielsen, M. (2020). Netmhcpan-4.1 and netmhciipan-4.0: improved predictions of mhc antigen presentation by concurrent motif deconvolution and integration of ms mhc eluted ligand data. Nucleic Acids Res. *48*, W449–W454. https://doi.org/10.1093/nar/gkaa379.
34. Bassani-Sternberg, M., and Gfeller, D. (2016). Unsupervised hla peptidome deconvolution improves ligand prediction accuracy and predicts cooperative effects in peptide-hla interactions. J. Immunol. *197*, 2492–2499. https://doi.org/10.4049/jimmunol.1600808.
35. Sun, X., Shi, Y., Akahoshi, T., Fujiwara, M., Gatanaga, H., Schönbach, C., Kuse, N., Appay, V., Gao, G.F., Oka, S., and Takiguchi, M. (2016). Effects of a single escape mutation on t cell and hiv-1 co-adaptation. Cell Rep. *15*, 2279–2291. https://doi.org/10.1016/j.celrep.2016.05.017.
36. Wu, K., Yost, K.E., Daniel, B., Belk, J.A., Xia, Y., Egawa, T., Satpathy, A., Chang, H.Y., and Zou, J. (2021). Tcr-bert: learning the grammar of t-cell receptors for flexible antigen-xbinding analyses. bioRxiv *2021–11*. https://doi.org/10.1101/2021.11.18.469186.
37. Abramson, J., Adler, J., Dunger, J., Evans, R., Green, T., Pritzel, A., Ronneberger, O., Willmore, L., Ballard, A.J., Bambrick, J., et al. (2024). Accurate structure prediction of biomolecular interactions with alphafold3. Nature *630*, 493–500. https://doi.org/10.1038/s41586-024-07487-w.
38. Schober, K., Müller, T.R., Gökmen, F., Grassmann, S., Effenberger, M., Poltorak, M., Stemberger, C., Schumann, K., Roth, T.L., Marson, A., and Busch, D.H. (2019). Orthotopic replacement of t-cell receptor α- and β-chains with preservation of near-physiological t-cell function. Nat. Biomed. Eng. *3*, 974–984. https://doi.org/10.1038/s41551-019-0409-0.
39. Mateyka, L.M., Strobl, P.M., Jarosch, S., Scheu, S.J.C., Busch, D.H., and D'Ippolito, E. (2022). Gene signatures of t-cell activation can serve as predictors of functionality for sars-cov-2-specific t-cell receptors. Vaccines *10*, 1617. https://doi.org/10.3390/vaccines10101617.
40. Moosmann, C., Müller, T.R., Busch, D.H., and Schober, K. (2022). Orthotopic t-cell receptor replacement in primary human t cells using crispr-cas9-mediated homology-directed repair. STAR Protoc. *3*, 101031. https://doi.org/10.1016/j.xpro.2021.101031.
41. Müller, T.R., Schuler, C., Hammel, M., Köhler, A., Jutz, S., Leitner, J., Schober, K., Busch, D.H., and Steinberger, P. (2020). At-cell reporter platform for high-throughput and reliable investigation of tcr function and biology. Clin. Transl. Immunology *9*, e1216. https://doi.org/10.1002/cti2.1216.
42. Kozak, M. (1987). An analysis of 5'-noncoding sequences from 699 vertebrate messenger RNAs. Nucleic Acids Res. *15*, 8125–8148. https://doi.org/10.1093/nar/15.20.8125.
43. Purcarea, A., Jarosch, S., Barton, J., Grassmann, S., Pachmayr, L., D'Ippolito, E., Hammel, M., Hochholzer, A., Wagner, K.I., van den Berg, J.H., et al. (2022). Signatures of recent activation identify a circulating t cell compartment containing tumor-specific antigen receptors with high avidity. Sci. Immunol. *7*, eabm2077. https://doi.org/10.1126/sciimmunol.abm2077.
44. Dössinger, G., Bunse, M., Bet, J., Albrecht, J., Paszkiewicz, P.J., Weißbrich, B., Schiedewitz, I., Henkel, L., Schiemann, M., Neuenhahn, M., et al. (2013). Mhc multimer-guided and cell culture-independent isolation of functional t cell receptors from single cells facilitates tcr identification for immunotherapy. PLoS One *8*, e61384. https://doi.org/10.1371/journal.pone.0061384.
45. Nauerth, M., Weißbrich, B., Knall, R., Franz, T., Dössinger, G., Bet, J., Paszkiewicz, P.J., Pfeifer, L., Bunse, M., Uckert, W., et al. (2013). Tcr-ligand k off rate correlates with the protective capacity of antigen-specific cd8+ t cells for adoptive transfer. Sci. Transl. Med. *5*, 192ra87. https://doi.org/10.1126/scitranslmed.3005958.
46. Lückemeier, P., Molter, K.L., Jarosch, S., Huppertz, P., Purcarea, A., Effenberger, M.J.P., Nauerth, M., D'Ippolito, E., Schober, K., and Busch, D.H. (2022). Global koff-rates of polyclonal t-cell populations merge subclonal avidities and predict functionality. Eur. J. Immunol. *52*, 582–596. https://doi.org/10.1002/eji.202149597.
47. Edgar, R.C. (2004). MUSCLE: multiple sequence alignment with high accuracy and high throughput. Nucleic Acids Res. *32*, 1792–1797. https://doi.org/10.1186/1471-2105-5-113.
48. Probst, P., Wright, M.N., and Boulesteix, A.-L. (2019). Hyperparameters and tuning strategies for random forest. WIREs Data Min. Knowl. *9*, e1301. https://doi.org/10.1002/widm.1301.
49. Jeni, L.A., Cohn, J.F., and De La Torre, F. (2013). Facing imbalanced data-recommendations for the use of performance metrics. In 2013 Humaine association conference on affective computing and intelligent interaction, pp. 245–251. https://doi.org/10.1109/ACII.2013.47.
50. Tickotsky, N., Sagiv, T., Prilusky, J., Shifrut, E., and Friedman, N. (2017). Mcpas-tcr: a manually curated catalogue of pathology-associated t cell receptor sequences. Bioinformatics *33*, 2924–2929. https://doi.org/10.1093/bioinformatics/btx286.
51. Schrödinger, L.L.C. (2015). The PyMOL molecular graphics system. Software version 1.8. .

STAR★METHODS

KEY RESOURCES TABLE

REAGENT or RESOURCE	SOURCE	IDENTIFIER
Antibodies		
aHLA-A*02 PE	BioLegend	RRID: AB_1877227
aHLA-B*07 PE	BioLegend	RRID: AB_2650774
aTCRβ APC	BioLegend	RRID: AB_313435
aCD3 Pacific Blue	BD Biosciences	RRID: AB_397038
aCD8 PC7	eBioscience	RRID: AB_2637437
Chemicals, peptides, and recombinant proteins		
Propidium Iodide (PI)	Life Technologies	Cat# P1304MP
RetroNectin®	Takara Bio Europe	Cat# T100B
Phorbol 12-myristate 13-acetate (PMA)	Sigma Aldrich	Cat# 16561-29-8
Ionomycin	Sigma Aldrich	Cat# 56092-81-0
RPMI 1640 Gibco	Sigma Aldrich	Cat# R0883
DMEM	Life Technologies	Cat# 10938025
Fetal calf serum	Biochrom	N/A
Gentamicin	Life Technologies	Cat# 15750-037
L-Glutamine	Sigma-Aldrich	Cat# G8540-100G
Penicillin/Streptomycin	Life Technologies	Cat# 10378016
Critical commercial assays		
SE Cell Line Kit	Lonza	Cat#: V4SC-1096
Experimental models: Cell lines		
Jurkat triple parameter reporter cells	In house	N/A
RD114	In house	N/A
K562 - HLA-A*02:01 - BFP	In house	N/A
K562 - HLA-B*07:02 - BFP	In house	N/A
Recombinant DNA		
MP71 vector for retrovirus generation in RD114 cells	Addgene	#108214
Software and algorithms		
FloJo V10	FlowJo LLC	https://www.flowjo.com/
Prism 9	Graphpad	https://www.graphpad.com
Python	Conda	Conda: python = 3.8
Numpy	Conda	Conda: numpy = 1.20.2
Pandas	Conda	Conda: pandas = 1.2.5
SciKit Learn	Conda	Conda: scikit-learn = 0.24.2
SciPy	Conda	Conda: scipy = 1.6.2
Generated Datasets	In house	Zenodo: https://doi.org/10.5281/zenodo.11195946
Custom Code	In house	Zenodo: https://doi.org/10.5281/zenodo.11197941
Other		
H-2K^{b}/mβ2m/Ova 257–264 Streptavidin- APC/BV421	Busch et al. (https://doi.org/10.1016/S1074-7613(00)80540-3)	N/A
HLA B*07:02/hβ2m/RNF43fs 273–283 Streptavidin- APC/BV421	Busch et al. (https://doi.org/10.1016/S1074-7613(00)80540-3)	N/A
HLA A*02:01/hβ2m/pp65 495–503 Streptavidin- APC/BV421	Busch et al. (https://doi.org/10.1016/S1074-7613(00)80540-3)	N/A

RESOURCE AVAILABILITY

Lead contact

Further information and requests for resources and reagents should be directed to and will be fulfilled by the lead contact, Benjamin Schubert (benjamin.schubert@helmholtz-munich.de).

Materials availability

This study did not generate new unique reagents.

Data and code availability

- The murine and two human datasets, the aligned TCR sequences, TCR distances, and the structural models were deposited at Zenodo: https://doi.org/10.5281/zenodo.11197941 and are publicly available as of the date of publication and are additionally included in the supplementary material. The DOI is listed in the key resources table.
- All code including all experiments to reproduce the results, analysis, and tutorials has been deposited at Zenodo: https://doi.org/10.5281/zenodo.11197941 and GitHub: https://github.com/SchubertLab/TcrPrediction_MutatedAPLs as of the date of publication. The DOI is listed in the key resources table.
- Any additional information required to reanalyze the data reported in this paper is available from the lead contact upon request.

EXPERIMENTAL MODEL AND STUDY PARTICIPANT DETAILS

Cell lines

RD114 cell line was grown in Dulbeccos Modified Eagle Medium, supplemented with 10% FCS, 0.025% L-Glutamine, 0.1% HEPES, 0.001% gentamycin and 0.002% streptomycin (cDMEM). K562 and JTPR were grown in Roswell Park Memorial Institute medium, supplemented with 10% FCS, 0.025% L-Glutamine, 0.1% HEPES, 0.001% gentamycin and 0.002% streptomycin (cRPMI). All cells were grown in a 37°C humidified, 5% CO2 incubator. JTPR were originally obtained from Peter Steinberger (Medizinische Universität Wien).

METHOD DETAILS

CRISPR-Cas9-mediated orthotopic TCR replacement

CRISPR-Cas9-mediated orthotopic TCR replacement (OTR) for RNF43-specific TCRs was performed as previously described.[38–40] All HDR templates were constructed in the following manner: *5′* homology arm (human TCR alpha chain constant region), P2A, TCRβ, T2A, TCRα, bovine growth hormone polyadenylation signal (pGHpA), *3′* homology arm. All TCR constructs were assembled in silico and synthesized in an ampicillin expression vector by Twist Bioscience. The dsDNA HDR templates were generated by PCR as previously described.[38,40] For the generation of assembled guide RNA (gRNA) of human TCRα constant (hTRAC) and human TCRβ constant region (hTRBC) (40μM), equal amounts of crRNA (80μM) was annealed with tracrRNA (80μM) at 95°C for 5min. Subsequently, 6μM Cas9 (61μM) was combined with the respective gRNAs and incubated for 15 min at RT to generate ribonucleoproteins (RNPs). For the electroporation procedure, 1μg of template DNA (1 μg/μL) was co-incubated for at least 30s with RNPs. JTPR cells were resuspended in 20μL SE buffer with added supplement (18μL/100μL) and mixed with the assembled RNPs for nucleofection. The electroporated JTPR cells (hTRBC, hTRAC, HDR template) were then transferred to 96-well U-bottom plates containing 175μL cRPMI without antibiotics and transferred to cRPMI with antibiotics after 24h.

Generation of retroviruses and transduction

The TCR DNA templates were designed in silico based on retrieved TCR sequences from single cell PCR. TCR constructs were synthesized by Twist Bioscience in a retroviral vector. The pp65-reactive DNA constructs had the following structure as previously described[41]: TCRβ chain including mTRBC1 (Ensembl: ENSMUST00000192856.6), P2A, TCRα chain, including hTRAC (Ensembl: ENSG00000277734.8). SIINFEKL-reactive TCRs had the following structure as previously described[14]: Murine Kozak sequence,[42] TCRβ chain including mTRBC1 (Ensembl: ENSMUST00000192856.6), P2A, TCRα chain, including mTRAC (Ensembl: ENSMUST00000103740.2). All TCRs were cloned into the pMP71 vector (kindly provided by Wolfgang Uckert, Berlin, added as Addgene plasmid backbone #108214). For retrovirus production, RD114 packaging cells were transfected with the retroviral vectors encoding for pp65-reactive TCRs via calcium phosphate precipitation. The supernatant of RD114 cells was collected at 72h after transfection and purified from remaining cells by centrifugation at 1,500r.p.m. at 4°C for 7min. The supernatant was stored at 4°C and used within 4 weeks after collection. Non-treated 48-well plates were coated with 120μL of RetroNectin (1:100 in PBS) over night at 4°C. After incubation the remaining PBS was removed and 400μL of RD114 virus supernatant encoding a specific TCR was added per well of a tissue-culture treated 48-well plate and centrifuged at 3,000 x g at 32°C for 2 h. After centrifugation, 350μL the virus supernatant was removed. 40,000 JTPR were added in 400μL of cRPMI to each coated well. The cells were centrifuged for 15 min at 800 x g at 32°C and incubated (37°C, 5% CO2) for 48 h. Transduction efficacy was determined via flow cytometry and transduced cells were purified by fluorescence-activated cell sorting for comparable TCR expression.

Data collection

Murine dataset

In this work, we analyzed the dataset described in Straub et al.[14] The authors experimentally determined TCR functional reactivity in response to mutations of the SIINFEKL epitope presented on the H-2K^b allele. Each epitope residue at every position was exchanged against all other 19 encoded amino acids, at a time, resulting in a library of 152 unique mutations of the wild-type peptide. Functional reactivity against these APLs was experimentally determined for 36 different murine TCRs as described by Straub et al.[14] In brief, Jurkat triple parameter reporter cells (JTPRs) were engineered to express a single SIINFEKL-reactive TCR, and co-incubated with peptide-pulsed splenocytes. After 24h incubation time, NFAT reporter expression was assessed via flow cytometry. The murine TCR library consisted of 15 unique TCRs isolated from the memory compartment of mCMV-SIINFEKL infected C57Bl/6 mice (educated repertoire, Data S1),[15,43] as well as 20 SIINFEKL-reactive TCRs from a naive C57Bl/6 donor (naive repertoire, Data S2).[14] The TCR OT-I was included as a reference control. TCR sequences were isolated from single-cell sorted, H-2K^b/SIINFEKL multimer positive CD8$^+$ T cell clones stemming from either infected or naive donors via the TCR SCAN platform[44] as described by Straub et al. TCR functional reactivity as assessed by JTPR stimulation was normalized as an activation score *A* across experiments (Datas S1 and S2). JTPRs expressing a unique TCR were stimulated with the APL library in independent experiments resulting in $NFAT_{\mathrm{APL}}$. In order to normalize the data, JTPRs of each TCR were included simultaneously in a single experiment and stimulated with the wild-type peptide resulting in $NFAT_{sim}$. $\mathrm{NFAT_{APL}}$ expression from APL library stimulated JTPR were normalized to this experiment:

$$A[APL] = NFAT_{APL} * \frac{NFAT_{sim}[SIINFEKL]}{NFAT_{APL}[SIINFEKL]} \quad \text{(Equation 1)}$$

For computational analysis to predict meaningful T cell activation, we set a threshold in the activation score of 46.9%. As described by Straub et al., this value was experimentally determined in this screening platform to predict effective recruitment and clonal expansion *in vivo* after adoptive transfer of low numbers of TCR transgenic naive T cells and infection. For the predictions, we excluded 11 TCRs for which the activation scores of all APLs fall short of this threshold.

Neo-epitope dataset

This dataset was experimentally generated in an analogous manner to the murine dataset. The APLs were formed by every single-amino acid mutation of the human cancer neo-epitope VPSVWRSSL. Prior to the experiments, binding of the APLs to the HLA-B*07:02 allele was computationally determined via NetMHCpan 4.1.[33] APLs without predicted binding were excluded from the dataset affecting positions P1, P2, P6, and P9 with 2, 19, 2, and 15 mutations, respectively, leading to a total amount of 133 peptides. JTPRs were engineered to express a single neo-epitope specific TCR recognizing the VPSVWRSSL epitope. JTPRs were co-incubated with peptide-pulsed K562 cells expressing HLA-B*07:02 for 24h. After 24h incubation time, NFAT reporter expression was assessed via flow cytometry. The repertoire consists of 7 TCRs isolated via pMHC multimer staining and antibody staining for a naive phenotype (CD3$^+$ CD8$^+$ CD45RA$^+$ CD62L$^+$) from healthy donors. In all experiments, the TCR R27 was excluded as it did not show the expected activation against all APLs. The percentage of activated cells was experimentally determined for the remaining combinations leading to a total of 798 pMHC-TCR interactions. The activation scores were normalized by their positive control. 66.09% was chosen as a threshold for binarization, which represents the lowest activation of a TCR against the cognate epitope alongside suboptimal tumor-cell lysis *in vitro* (data not shown, Data S3).

CMV dataset

These data were generated in the same manner as described for human neo-epitope reactive TCRs. The APLs were formed by single-amino acid mutations of the human CMV HLA-A*02:01 restricted epitope NLVPMVATV (pp65), irrespective of binding prediction to the HLA allele, leading to a total amount of 172 peptides. JTPRs were engineered via retroviral transduction to express a single TCR recognizing the pp65 epitope. The pp65 TCR library comprised 20 unique TCRs derived from CMV seropositive donors that were previously isolated and provided by Mueller and colleagues.[41] JTPR were co-incubated with peptide-pulsed K562 cells expressing HLA-A*02:01 and NFAT reporter activation was assessed as described. In total, we measured 3,440 unique pMHC-TCR interactions. We chose a threshold of 40.0% for binary classification of antigen recognition. This threshold represents the lowest activation of a TCR against the pp65 epitope which displayed a detectable TCR-ligand k_{off} rate (data not shown). This TCR affinity measurement is indicative of high functionality and underscores a significant binding strength to the epitope.[45,46] JTPR were stained for surface antigens (TCR β-chain, CD3) to assess the percentage of TCR transgenic cells. The activation scores were normalized based on the fraction of transgenic TCR-expressing JTPR (Data S4).

Predictors

Data representation

To provide the Random Forests with information on the APLs, we encoded their sequences into numeric representations. To this end, we represent each amino acid via five factors representing a summary of physiochemical properties as developed by Atchley et al.[17] which summarize polarity, secondary structure, molecular size, amino acid composition in proteins, and electrostatic charge. Based on this encoding, we provided the full APL sequence and the difference between the APL and the wild-type sequence. Additionally, the position of the mutation, the original, and the new amino acid at this position are provided.

When predicting across TCRs, we provided the CDR3 sequences of the α- and β-chain as additional input to the Random Forests. To this end, we represented the amino acid of each position via the Atchley factors as described above. To counteract the effect of different lengths, we aligned the sequences using the Multiple Sequence Comparison by Log-Expectation (MUSCLE) algorithm[47] for the murine and two human datasets, separately (Data S5), which aligns multiple biological sequences by introducing padding while still preserving homology. Padding tokens were consequently encoded with all zero values.

Random Forests

The Random Forest predictors were consequently trained on this representation. While each Random Forest consisted of 250 different Decision Trees when predicting activation for the remaining APLs of a TCR, the number of Decision Trees was increased to 1,000 for cross-TCR prediction to tackle the larger feature space. Each individual tree was fit on a bootstrap sample of the data and a random subset of $\sqrt{d}$ features. We chose this setup with a large amount of diverse trees to prevent overfitting and aid generalization,[48] and the resulting performance is saturated by the number of trees (Figure S20). The trees were fully grown using the Gini impurity as the splitting criterion in case of classification and mean absolute error for regression, in order to avoid outliers dominating the models' predictions.

Metrics

The classification models were mainly evaluated by the AUC and APS. The AUC can be interpreted as the probability that a positive sample is scored higher than a negative sample, and is thus a natural and common metric used in binary classification tasks. The APS on the other hand, is more robust toward imbalanced data, thus providing a reliable performance measure even when most events are negative, as we observed for some TCRs in the dataset.[49] To avoid differences in activation scores caused by the normalization, we used the Spearman's rank correlation to evaluate regression models. To calculate the accuracy, the prediction was binarized at a threshold of 50% classification probability, which signifies the predicted likelihood of an APL exceeding the dataset-dependent activation score threshold.

Perturbation tests

To determine the importance of input features, the Random Forest was first trained as described above on the unperturbed data. Following, selected groups of input features – but not the labels – were randomly shuffled. Intuitively, using random values for features breaks their dependency on the target. Therefore, the model is not able to predict accurately when important input features are perturbed and thus the model's performance is greatly reduced. Unimportant features, instead, are not used by well-performing models, thus using random values for them should not impact performance. For P-TEAM, either the full CDR3 region or the amino acid at each position individually was perturbed. In the former case, the model gets presented with the sequence of a random other TCR, but is evaluated on the original TCR. Therefore, a drop in performance indicates that the model strongly relies on the CDR3 sequence for its prediction and does not solely reproduce the result of a random other TCR.

Baseline TCR predictors and distances

The data for the TCR predictors ERGO-II[8] and ImRex[10] were formatted as described by the authors in the corresponding GitHub repositories. While the trained model provided for ImRex uses the CDRβ sequence as a sole input, ERGO-II can optionally incorporate the CDR3α sequence, V- and J-genes of both chains, and MHC type. To allow fair comparison, we reduced the input for ERGO-II to the information used by P-TEAM, i.e., the sequences of the CDR3 sequences of both chains. ERGO-II offers two different models which were trained on the VDJdb[12] and McPas-TCR[50] databases, respectively. Since it is unclear which model better fits the data used in this work, we report the performance of both models. Distances between the TCRs within a dataset were calculated based on the implementation of TCRdist3[20] (Data S6). To derive epitope similarities, we compared the amino acid of the base epitope and its mutation in the APL. The amino acid similarity was determined by the corresponding entry in the BLOSUM62 matrix[22] or by the Euclidean distance of their Atchley factors,[17] which was subtracted from 10 to convert the distance to similarity. For the epitope similarity measurements, the threshold was set per position to optimize the corresponding accuracy.

Structural modeling

The full nucleotide sequences of the TCRα and TCRβ chains (Datas S1–S4) were translated to amino acid sequences. α- and β-chains of each TCR, the wild-type epitope and the sequence of the MHC (H-2K^b for SIINFEKL, HLA-B*07:02 for VPSVWRSSL, HLA-A*02:01 for NLVPMVATV) were used as input for TCRpMHCmodels-1.0[25] to derive the structural models (Datas S7–S9). Four TCRs of the educated repertoire (TCR ED5, TCR ED10, TCR ED23, and TCR ED40) were excluded from the following tasks, as the modeling software failed to derive structural models presumably due to lack of TCR templates for these sequences. These models were aligned by their MHC and visualized with PyMol,[51] which also served as an interface to determine the structural relationships. The distances of the center of mass for each amino acid residue in the CDR3α and CDR3β toward all peptide residues were calculated via the 'center-ofmass' command. Following, the minimal distances between TCR and epitope were determined for each epitope position.

Active learning

Active learning was used to reduce the amount of data needed to derive accurate predictors by choosing training samples in a sophisticated manner. We applied active learning in two settings. First, the algorithm selected the best APLs for an individual TCR to predict the remaining APLs. Second, given a set of TCRs for which the activation score was known for all APLs, the algorithm selected

the best APLs to be experimentally determined for a novel TCR. The general workflow of the active learning procedure followed an iterative approach (Algorithm 1). In our experiments, we simulated the iterative experimental procedure by holding out the activation scores for all yet unknown samples.

Initialization

The activation scores A_{init} were experimentally determined for an initial set of training samples S_{init} out of the full set of samples S_{full}. This initial set consisted of the APLs with the largest BLOSUM62[27] distance to the base epitope for each position and the wild-type epitope itself. These S_{init} and A_{init} were assigned as training samples S_{train} and training activation labels A_{train}.

Iterative process

After initialization, the iterative process is started. A classification predictor following the same model as described above was trained on S_{train} and A_{train}. This classifier predicted the binary activation for the remaining APLs ($S_{full} \setminus S_{train}$). In each step, the N_{add} APLs S_{new} with the most uncertain prediction were identified and the corresponding activation scores A_{new} were experimentally determined. Following, S_{new} and A_{new} were added to S_{train} and A_{train}, respectively. After the evaluation of the yet unobserved data, the iterative process continued with this updated training set until $M=10$ iterations were reached.

Uncertainty

This active learning process requires a measure of prediction uncertainty for each sample. Since the Random Forest consists of an ensemble of different Decision Tree classifiers, the proportion of votes between these individual predictors can be interpreted as the class probability of the Random Forest. Since the models were biased toward the dominating class of the training set, the inverse difference of this class probability for each sample toward the average class probability across all samples was used to indicate the uncertainty of the model.

Evaluation

At each iteration, the predictors were tested for classification as well as regression based on the selected samples S_{train}. For this evaluation, the unobserved APLs ($S_{full} \setminus S_{train}$) were used. The active learning scheme was compared against a baseline model, for which training data was added randomly. The experiments were conducted on 100 random seeds to obtain robust performance estimates for the different acquisition methods.

QUANTIFICATION AND STATISTICAL ANALYSIS

All statistical analyses were performed in Python (version 3.8) using the libraries SciKit Learn (version 0.24.2), SciPy (version 1.6.2), Numpy (version 1.20.2), and Pandas (version 1.2.5). The sample size n and its description can be found in the corresponding figure legends. All boxplots indicate the data quartiles while the whiskers extend to the extreme values excluding outliers outside the 1.5 interquartile range. The median is indicated as a horizontal line. If present, a triangle highlights the mean. Regression plots show the linear regression fit and line plots the mean of the data as a line, and both indicate the 95% confidence interval as an error band. Bar plots represent the data mean and their error bars the 95% confidence interval. Significance was defined by a p-value less than 0.05 with the values indicated through the following symbols: * <0.05, ** <0.01, *** <0.001, **** <0.0001. If not stated otherwise in the corresponding main text, a two-sided, paired t test was used to compare performances. For Spearman and Pearson correlations, a t test against the null hypothesis that the data are uncorrelated was performed. The present study utilized all samples excluding eleven TCRs from the naive without reactivity to any of the presented APLs. Four TCRs of the educated repertoire (TCR ED5, TCR ED10, TCR ED23, and TCR ED40) were excluded from the distance calculation as the modeling software failed to derive their structural models.

Cell Genomics, Volume *4*

Supplemental information

Predicting T cell receptor functionality against mutant epitopes

Felix Drost, Emilio Dorigatti, Adrian Straub, Philipp Hilgendorf, Karolin I. Wagner, Kersten Heyer, Marta López Montes, Bernd Bischl, Dirk H. Busch, Kilian Schober, and Benjamin Schubert

Supplementary Figures

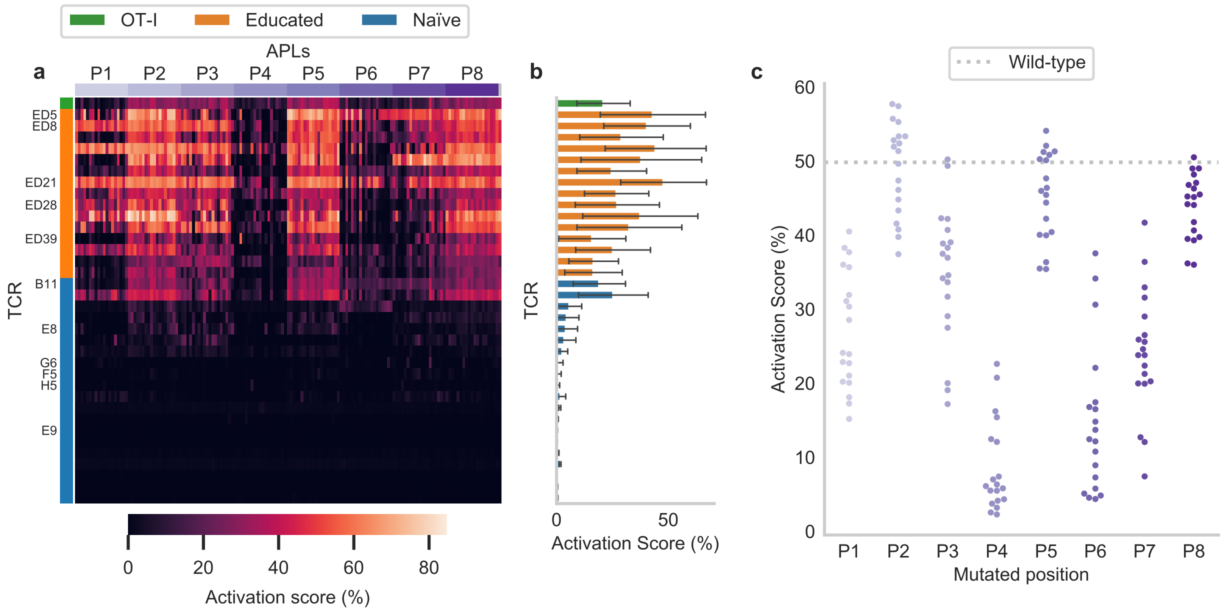

Figure S1 | Unnormalized T cell activation for the TCRs and APLs of the murine dataset, related to Figure 2. **a**, Unnormalized activation scores. **b**, Unnormalized activation scores averaged for all APLs and the wildtype epitope ($n = 153$) per TCR. **c**, Unnormalized activation per APL ($n = 19$) over all TCRs ($n = 36$).

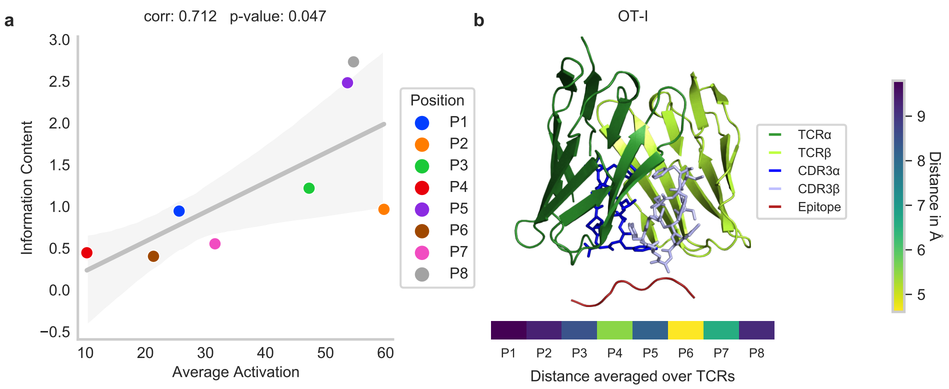

Figure S2 | Influence of MHC restrictions on the activation score, related to Figure 2. **a**, Correlation between the information content of the MHC motif positions measured in bits with the average activation scores for APLs mutated at these positions ($n = 8$) for H-2K^{b}. **b**, Structural model of OT-I/SIINFEKL predicted by TCRpMHCmodels [S1] and the distance of the residue to the nearest residue in the TCR averaged across the TCRs ($n = 32$) of the murine dataset.

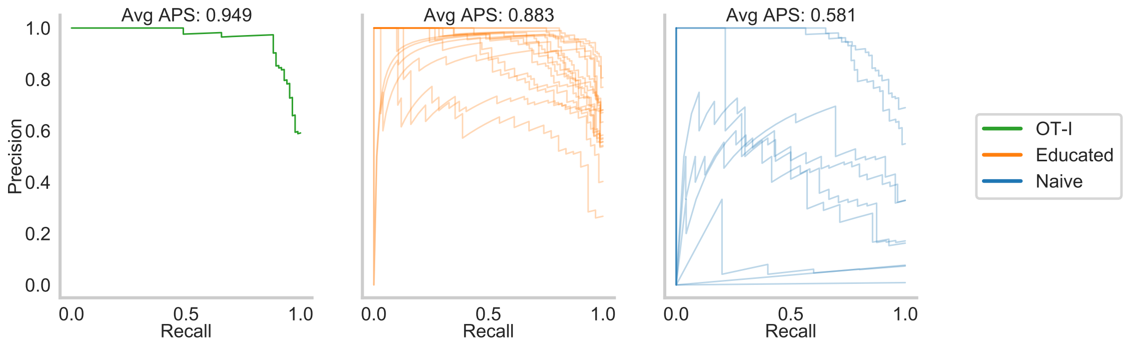

Figure S3 | Additional metrics for predicting within a TCR of the murine dataset, related to Figure 2. Precision-Recall curve with Average Precision Scores (APS) for the different groups of TCRs.

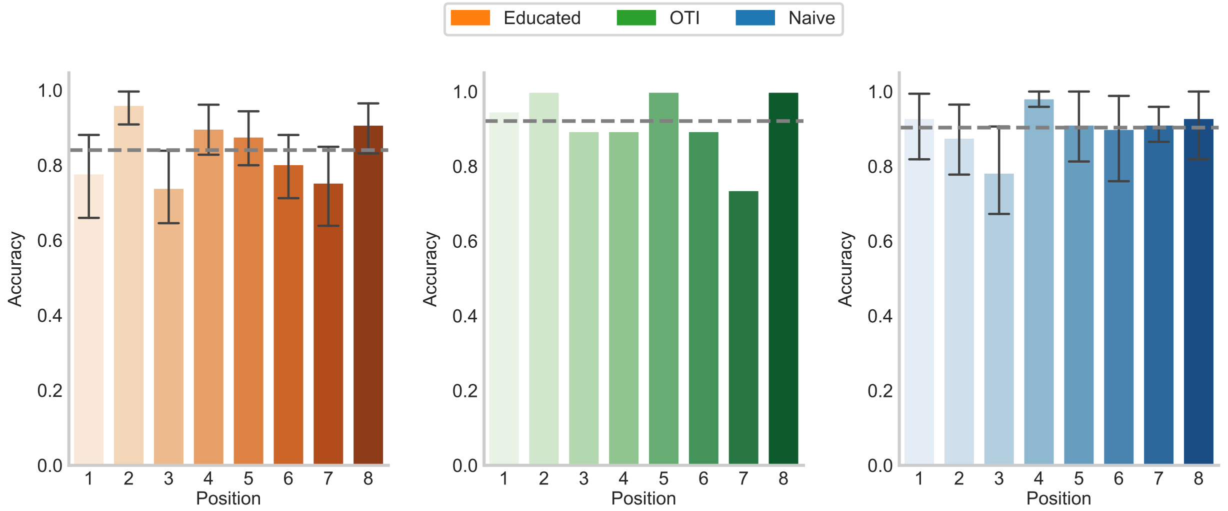

Figure S4 | Position-wise evaluation, related to Figure 2. Accuracy evaluated per position of mutation for the educated repertoire ($n = 15$), OT-I ($n = 1$), and the naive repertoire ($n = 9$) of the murine dataset. The dashed line shows the average position-wise accuracy over the whole dataset.

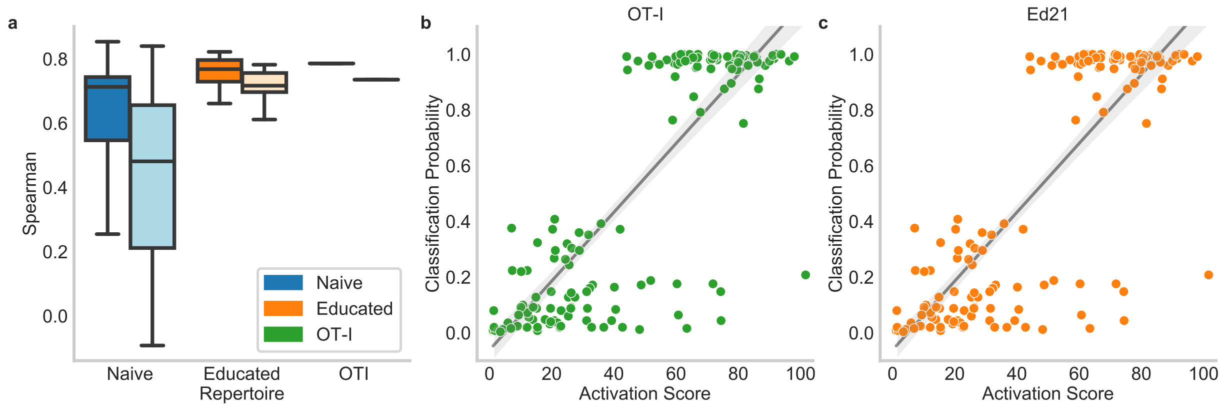

Figure S5 | Regression capacity of the classification model for the murine datasets, related to Figure 2. a, Regression evaluation of the regression model (dark color) compared to the classification model (light color) for the educated repertoire ($n = 15$), OT-I ($n = 1$), and the naive repertoire ($n = 9$) of the murine dataset. Exemplary correlation between activation score and classification probability of two representative TCRs: OT-I (**b**) and Ed21 (**c**) ($n = 152$ APLs).

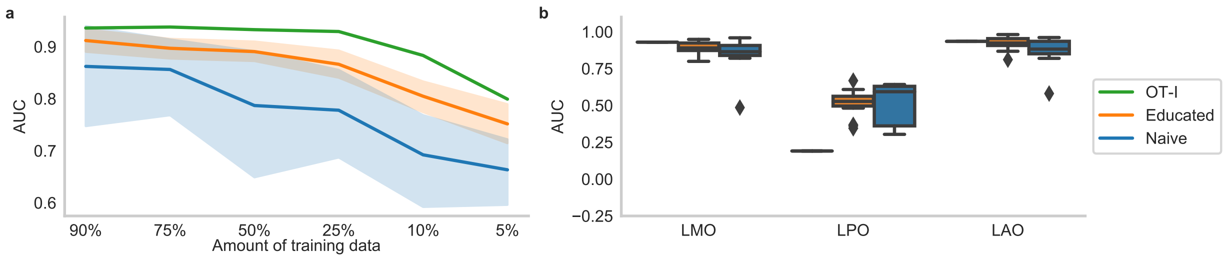

Figure S6 | Additional metrics for reducing the training data within a TCR of the murine dataset, related to Figure 2. a, AUC obtained when training on different subsets of the data. LMO: leave-mutation-out, LPO: leave-position-out, and LAO: leave-amino-acid-out. **b**, AUC when a smaller amount of training data is used (average over ten repetitions with random subsets for each TCR). The performance is shown for OT-I ($n = 1$ TCR), the educated repertoire ($n = 15$ TCRs), and the naïve repertoire ($n = 9$ TCRs).

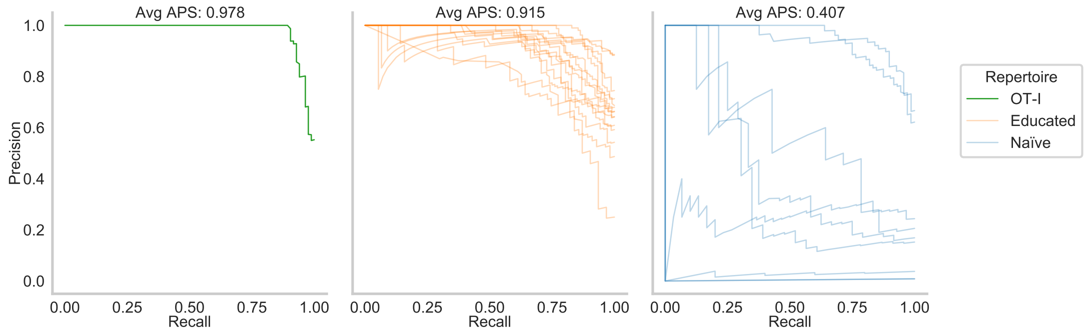

Figure S7 | Additional metrics for predicting across TCRs of the murine dataset, related to Figure 3. Precision-Recall curve with Average Precision Score (APS) for the different groups of TCRs.

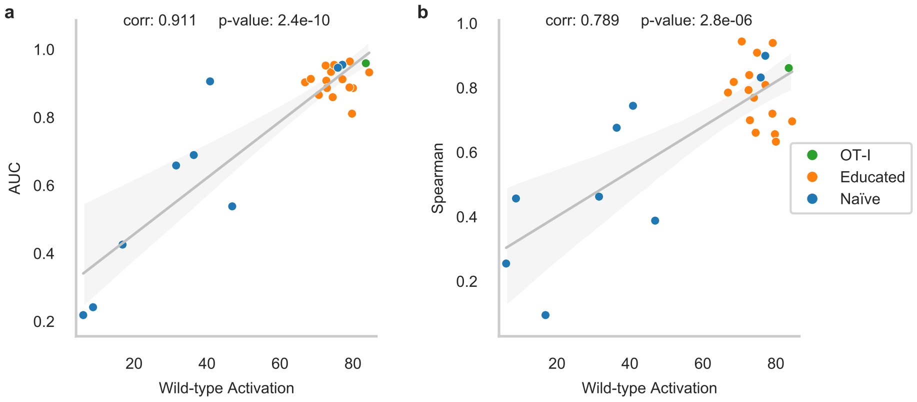

Figure S8 | Relationship between generalization performance and wild-type activation, related to Figure 3. The performance for classification (**a**) and regression (**b**) in the Leave-TCR-out setting shows a strong Pearson correlation to the normalized activation score against the wild-type epitope in the murine dataset ($n = 25$ TCRs).

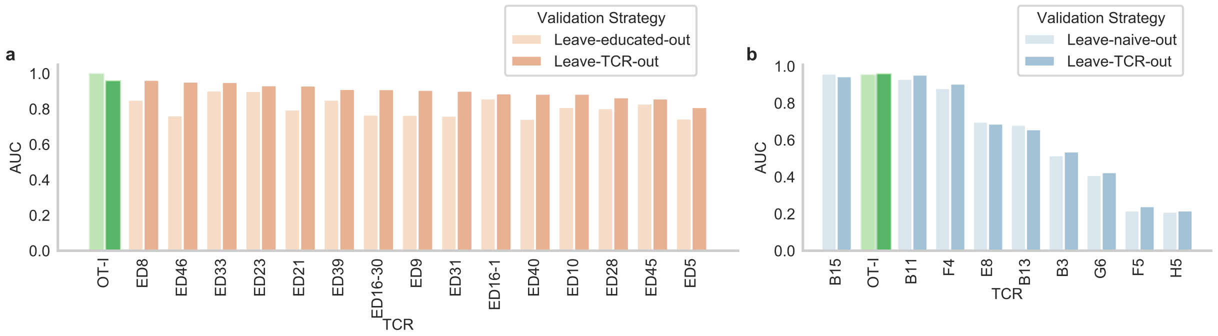

Figure S9 | Comparison of the AUC scores for training on different repertoires, related to Figure 3. **a**, Performance was evaluated on the 15 TCRs from the educated repertoire and OT-I when the model was trained on the full remaining murine dataset (Leave-TCR-out) or solely the TCRs of the naive repertoire (Leave-educated-out). **b**, Performance on the nine TCRs from the naive repertoire and OT-I for Leave-TCR-out training or training only on the TCRs from the educated repertoire (Leave-naive-out).

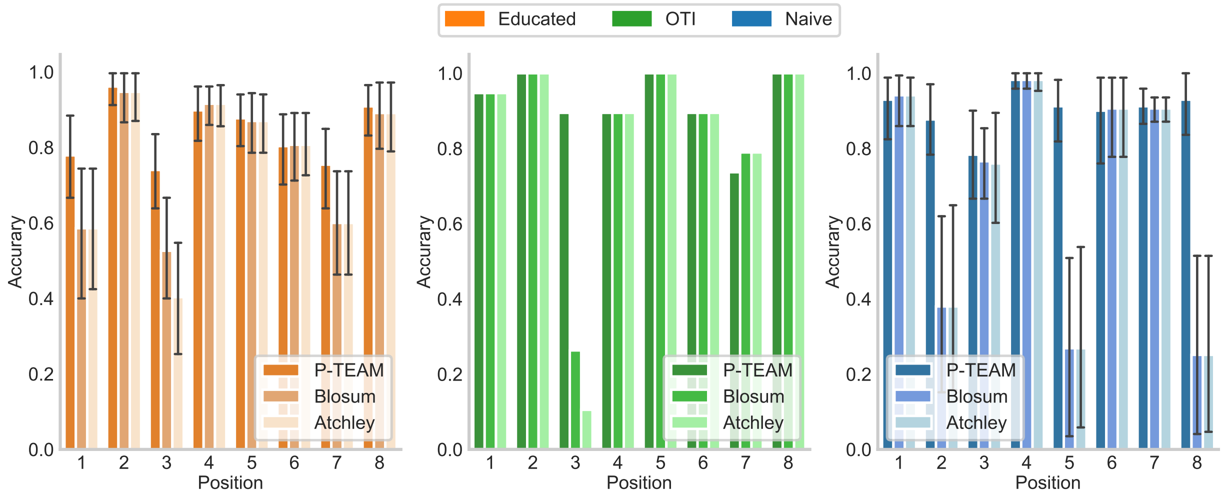

Figure S10 | Position-wise evaluation compared to peptide distances, related to Figure 3. Accuracy of P-TEAM evaluated per position of mutation compared to the amino acid distances of BLOSUM62 and Atchley factors for the educated repertoire ($n = 15$), OT-I ($n = 1$), and the naive repertoire ($n = 9$) of the murine dataset.

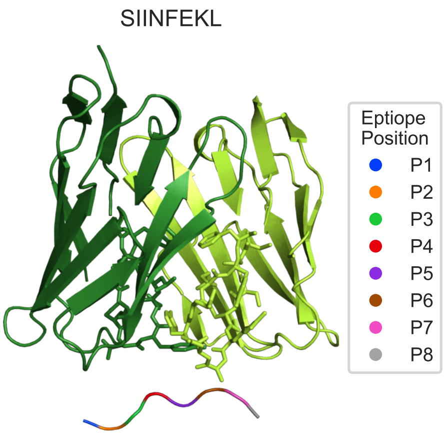

Figure S11 | Epitope positions in the structural model, related to Figure 3. Epitope positions are highlighted in the structural model for OT-I/SIINFEKL. TCRα and TCRβ chains are shown in dark and light green, respectively.

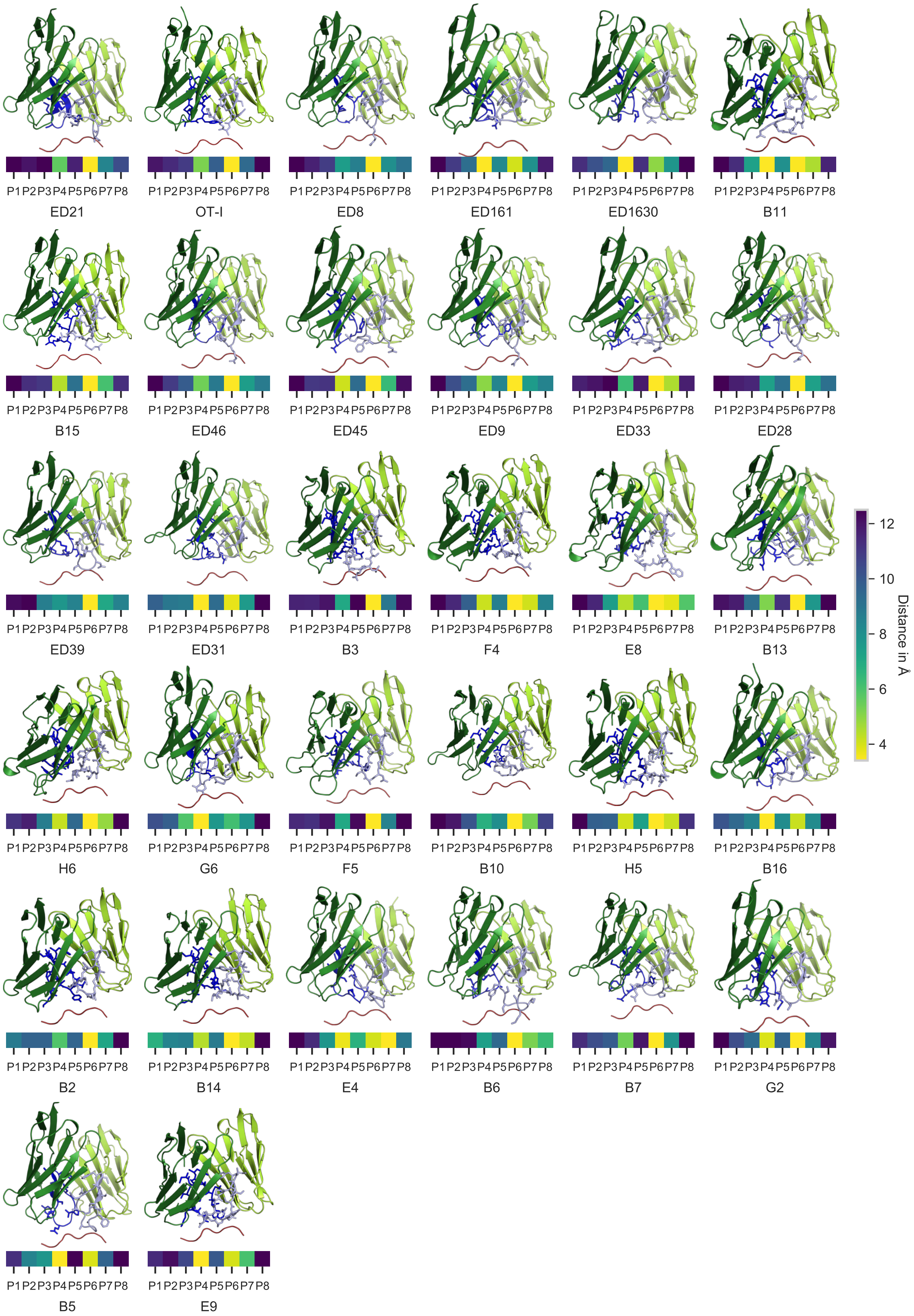

Figure S12 | Structural Models of the murine dataset, related to Figure 3. Predicted structures of the TCR and epitope, and minimal distance to the individual epitope positions for all receptors of the murine datasets ordered by descending activation to the wildtype epitope.

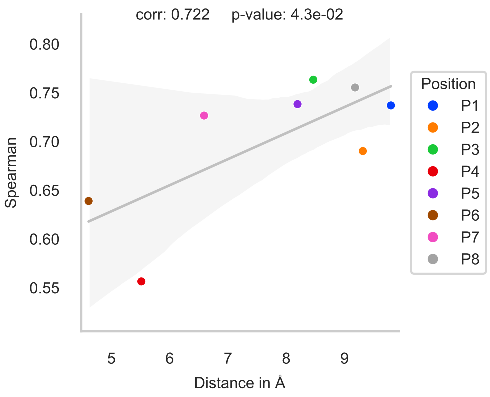

Figure S13 | Relationship between spacial distance and feature importance, related to Figure 3. The regression performance during perturbation tests at each epitope position shows strong Pearson correlation to the distance between this position and its closest TCR residue in the murine dataset (n = 8 positions).

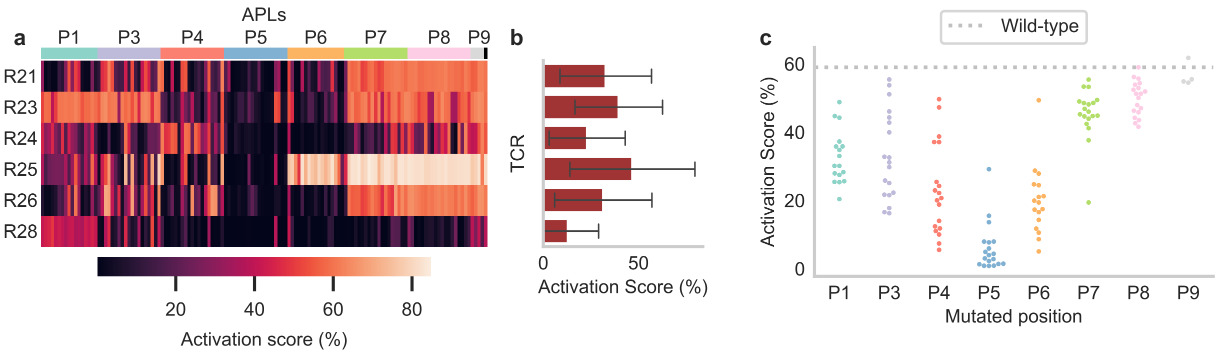

Figure S14 | Unnormalized T cell activation for the TCRs and APLs of the neo-epitope dataset, related to Figure 5. a, Unnormalized activation scores. **b**, Unnormalized activation scores averaged for all APLs ($n = 133$) per TCR. **c**, Unnormalized activation per APL over all TCRs ($n = 6$).

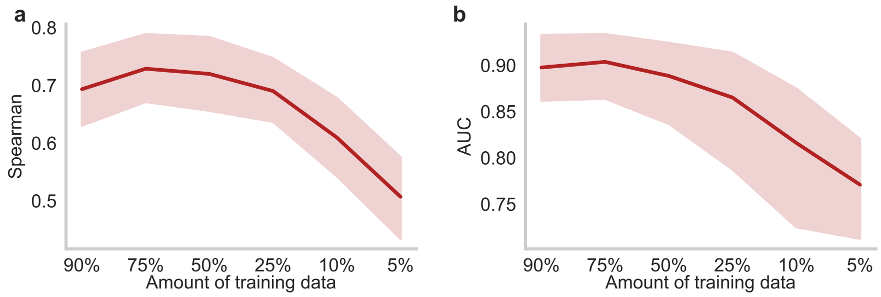

Figure S15 | Performance on the human dataset on limited training data, related to Figure 5. Spearman correlation (**a**) and AUC (**b**) when a smaller amount of training data is used ($n = 6$ TCRs $*$ 100 repetitions).

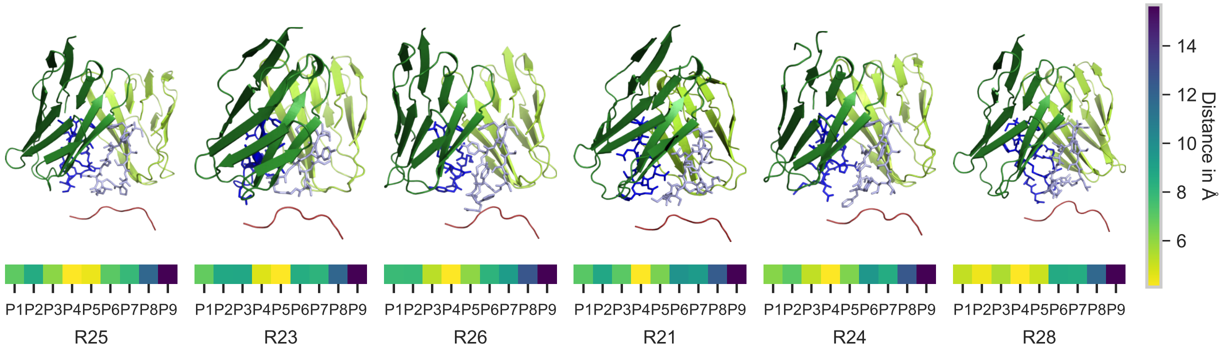

Figure S16 | Structural Models of the neo-epitope dataset, related to Figure 6. Predicted structures of the TCR and epitope, and minimal distance to the individual epitope positions for all receptors of the human datasets ordered by descending activation to the wildtype epitope.

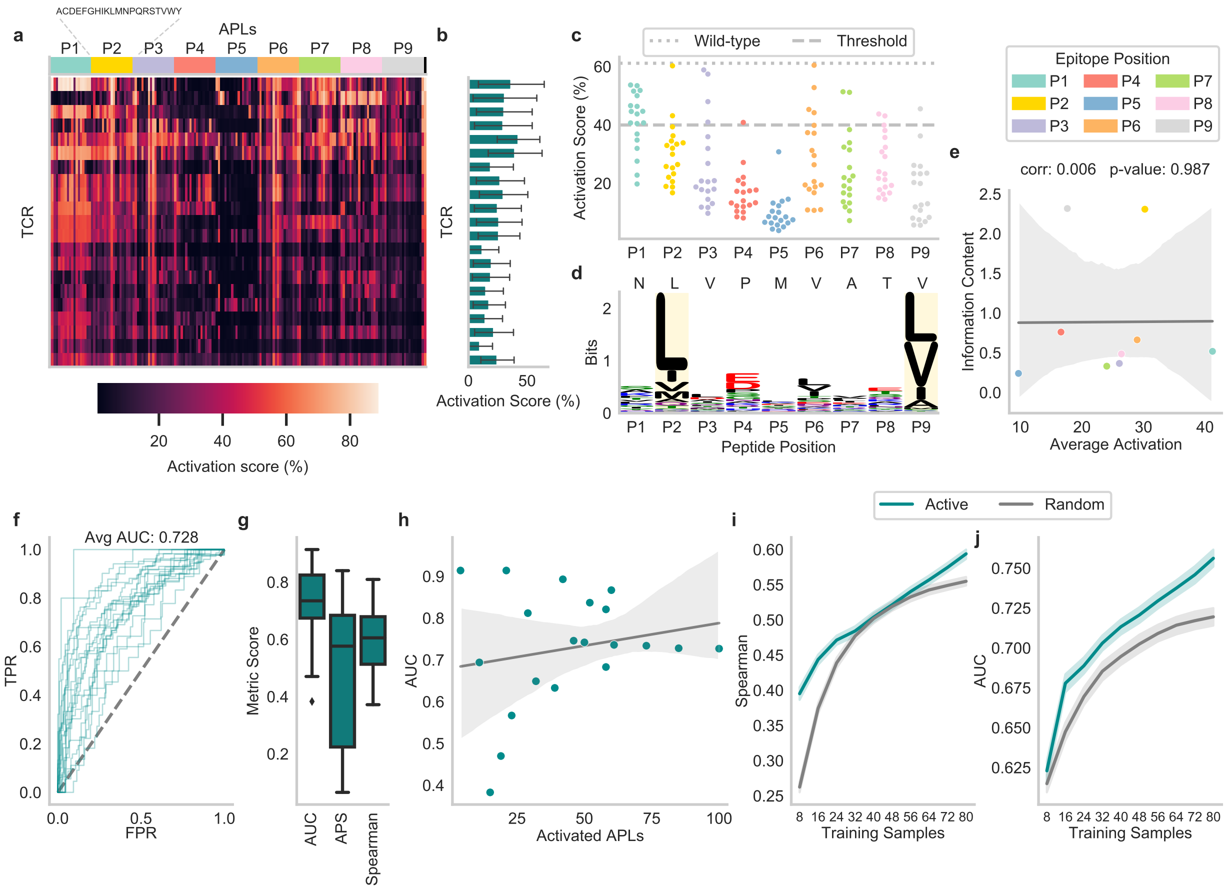

Figure S17 | Predicting the effect of mutations of a CMV epitope, related to Figure 2 and Figure 4. **a**, The normalized activation scores of 20 TCRs of the CMV dataset. **b**, The activation scores averaged for all APLs ($n = 171$) of one TCR. **c**, The epitope position on which the mutation occurs strongly influences the activation per APL ($n = 19$) averaged over all TCRs ($n = 20$). The threshold value represents the boundary between binding and non-binding and wild-type indicates the activation scores of the base epitope NLVPMVATV. **d**, MHC restrictiveness indicated by information content in bits for HLA-A*02:01 obtained from the MHC Motif Atlas [S2] per position from $n = 8,372$ peptides. Reported anchor positions are highlighted in yellow and the wildtype epitope is indicated above. **e**, Correlation between the Gini coefficient of the MHC motif positions with the average activation scores for APLs mutated at these positions for HLA-A*02:01 ($n = 9$ positions) **f**, The Receiver operating characteristic (ROC) curves of the CMV TCRs indicate the True Positive Rate (TPR) against the False Positive Rate (FPR) at all prediction values as thresholds. **g**, Different evaluation metrics for regression (Spearman) and classification models (APS: Average Precision Score, AUC: Area Under the ROC Curve). **h**, Performance of P-TEAM against the amount of activated APLs per TCR. The performance in e-g is shown for $n = 20$ TCRs. Comparison of the active learning framework to random sample selection on the CMV dataset for regression (**i**) and classification models (**j**). The expected performance is shown for up to $m = 10$ consecutive iterations ($N_{APLs} = 80$ APLs) of alternating wet lab experiments and model training ($n = 15$ TCRs $*$ 100 repetitions).

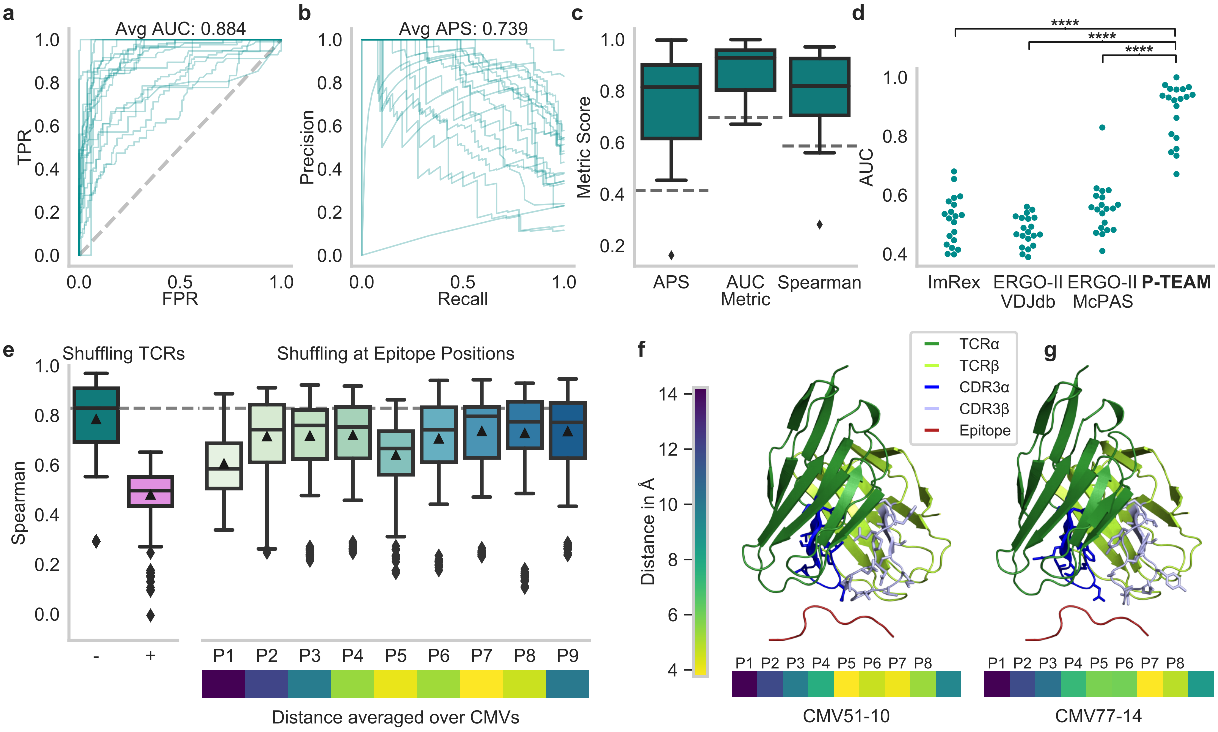

Figure S18 | Across-repertoire prediction for the CMV dataset, related to Figure 3. ROC (**a**) and Precision-Recall (**b**) curves for the 20 CMV-specific TCRs. **c**, Average precision score (APS), area under the ROC curve (AUC), and Spearman correlation as classification and regression metrics. The dashed line indicates the prediction using the labels of a random other TCR. **d**, P-TEAM outperforms existing TCR-epitope predictors ImRex [S3] and ERGO-II [S4] by a large margin (****: p-value < 0.0001). The performance in a-d is shown for $n = 20$ TCRs. **e**, The importance of input features obtained by replacing the test TCR input with a random CDR3 sequence of the dataset $(+)$ or by shuffling the amino acid at each epitope position in the test set compared to the un-shuffled performance (- and dashed line) ($n = 20$ TCRs $* 15$ repetitions). Below, the average distance of the center of mass between the epitope and TCR residues is shown. **f, g**, Predicted structural model of the TCR and epitope, and minimal distance to the individual epitope positions for receptors CMV51-10 and CMV77-14 (highest and lowest activation, respectively). The model shows the interaction between the epitope and the CDR3 of the TCRs.

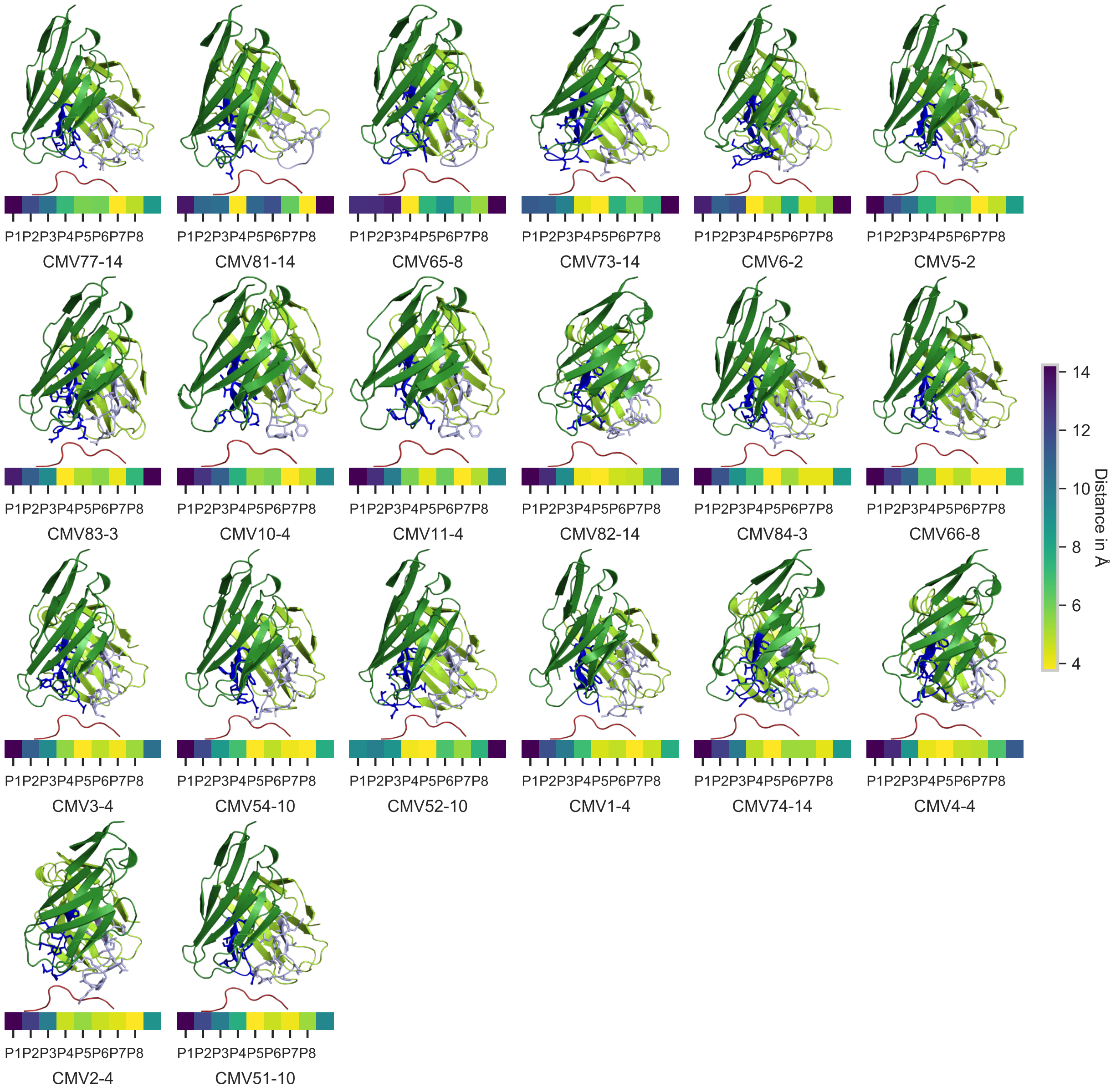

Figure S19 | Structural Models of the CMV dataset, related to Figure 3. Predicted structures of the TCR and epitope, and minimal distance to the individual epitope positions for all receptors of the human datasets ordered by descending activation to the wildtype epitope.

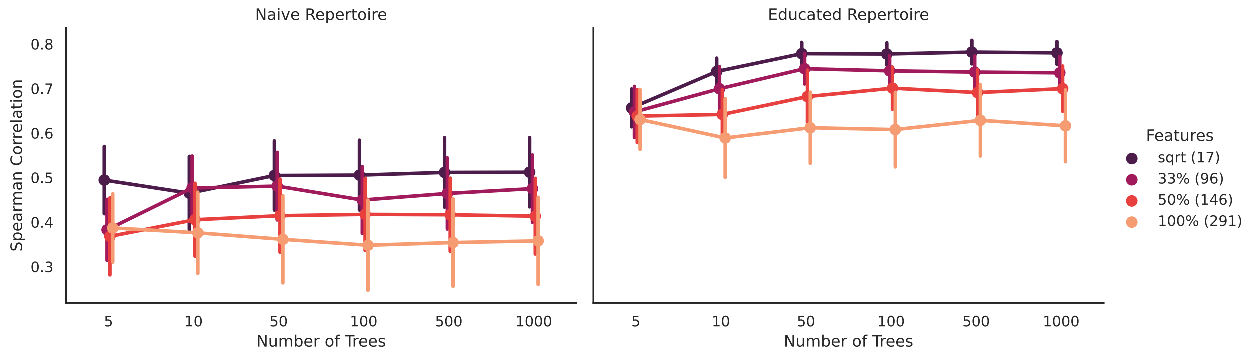

Figure S20 | Performance of P-TEAM by number of trees in the random forest, and number of features used to build each tree, related to STAR Methods. The Spearman correlation between the predicted and measured activation scores in a leave-TCR-out validation scheme is shown as a function of the number of trees used to train the random forests (x-axis), and the number of randomly-chosen features used to grow each tree.

Supplementary Tables

Repertoire	TCR	Leave-Mutation-Out AUC	Leave-Mutation-Out APS	Leave-Mutation-Out Spearman	Leave-TCR-out AUC	Leave-TCR-out APS	Leave-TCR-out Spearman
	OTI	0.930	0.949	0.786	**0.959**	**0.978**	0.861
Educated	ED5	0.907	0.946	0.746	0.811	0.880	0.656
	ED8	0.938	**0.980**	0.724	**0.965**	**0.990**	**0.939**
	ED9	0.875	0.900	0.772	0.908	0.938	0.840
	ED10	0.905	0.930	0.755	0.886	0.945	0.633
	ED16-1	**0.944**	0.946	0.802	0.888	0.928	0.720
	ED16-30	0.920	0.941	0.801	0.912	0.925	0.809
	ED21	**0.949**	**0.987**	0.709	0.933	**0.982**	0.696
	ED23	0.891	0.949	0.661	0.934	0.973	0.769
	ED28	*0.799*	0.668	0.735	0.866	0.807	**0.944**
	ED31	0.873	0.804	0.793	0.903	0.859	0.785
	ED33	0.928	0.908	0.708	0.953	0.923	0.793
	ED39	0.873	0.662	0.768	0.913	0.805	0.818
	ED40	0.850	0.829	**0.822**	0.886	0.895	0.700
	ED45	0.841	0.912	0.777	0.860	0.919	0.661
	ED46	0.890	0.880	**0.822**	0.955	0.960	**0.909**
Naive	B3	0.919	0.602	0.739	0.538	0.379	*0.388*
	B11	**0.959**	**0.954**	**0.854**	**0.955**	0.945	0.899
	B13	0.858	*0.521*	0.713	0.659	0.275	0.462
	B15	0.895	0.917	0.780	0.946	0.947	0.832
	E8	*0.821*	*0.447*	0.665	0.689	0.456	0.676
	F4	0.865	0.565	0.744	0.906	0.608	0.744
	F5	-	-	*0.547*	*0.242*	*0.009*	0.457
	G6	*0.486*	*0.062*	*0.255*	*0.426*	*0.034*	*0.095*
	H5	-	-	*0.307*	*0.219*	*0.008*	*0.255*

Table S1 | Performance of P-TEAM on the murine dataset, related to Figure 2 and Figure 3. The classification performance is reported by the area under the receiver operator characteristic curve (AUC) and the average precision score (APS). The regression performance is reported by the Spearman's rank coefficient. The three TCRs on which P-TEAM performed best and worst are shown in bold and italics, respectively, for each metric. AUC and APS of F5 and H5 could not be calculated as no sample was predicted as positive.

TCR	Leave-Mutation-Out AUC	APS	Spearman	Leave-TCR-out AUC	APS	Spearman
R21	0.906	0.882	0.682	**0.938**	**0.916**	**0.800**
R23	0.892	0.872	0.744	0.788	0.842	0.708
R24	*0.761*	*0.322*	0.700	0.618	0.275	*0.427*
R25	**0.949**	**0.933**	**0.846**	0.809	0.864	0.681
R26	0.920	0.901	0.780	0.916	0.899	0.766
R28	0.911	0.791	*0.654*	*0.561*	*0.183*	0.509

Table S2 | Performance of P-TEAM on the human dataset, related to Figure 5 and Figure 6. The classification performance is reported by the area under the receiver operator characteristic curve (AUC) and the average precision score (APS). The regression performance is reported by the Spearman's rank coefficient. The TCRs on which P-TEAM performed best and worst are shown in bold and italics, respectively, for each metric.

TCR	Leave-Mutation-Out AUC	APS	Spearman	Leave-TCR-out AUC	APS	Spearman
CMV4-4	**0.914**	0.780	0.775	**1.000**	**0.998**	**0.971**
CMV51-10	0.914	0.116	0.643	0.940	*0.163*	0.719
CMV82-14	0.893	0.709	0.775	0.757	0.707	0.959
CMV65-8	0.866	**0.840**	0.699	0.734	0.584	0.666
CMV84-3	0.838	0.672	**0.810**	0.927	0.871	0.899
CMV81-14	0.821	0.676	0.658	*0.671*	0.566	*0.282*
CMV54-10	0.812	0.556	0.616	0.937	0.801	0.830
CMV66-8	0.746	0.597	0.628	0.958	0.902	0.863
CMV10-4	0.742	0.540	0.595	0.974	0.916	0.966
CMV11-4	0.736	0.633	0.557	0.961	0.954	0.959
CMV77-14	0.734	0.655	0.692	0.922	0.901	0.853
CMV5-2	0.728	0.770	0.514	0.902	0.903	0.809
CMV6-2	0.727	0.796	0.373	0.794	0.846	0.632
CMV3-4	0.694	0.109	0.675	0.966	0.627	0.782
CMV73-14	0.683	0.437	0.526	0.931	0.893	0.931
CMV52-10	0.649	0.251	0.512	0.959	0.830	0.925
CMV2-4	0.633	0.331	0.540	0.862	0.710	0.798
CMV83-3	0.567	0.144	0.388	0.933	0.676	0.735
CMV74-14	0.470	0.098	*0.372*	0.745	0.454	0.561
CMV1-4	*0.383*	*0.065*	0.495	0.807	0.485	0.610

Table S3 | Performance of P-TEAM on the CMV dataset, related to Figure 2 and Figure 3. The classification performance is reported by the area under the receiver operator characteristic curve (AUC) and the average precision score (APS). The regression performance is reported by the Spearman's rank coefficient. The TCRs on which P-TEAM performed best and worst are shown in bold and italics, respectively, for each metric.

References

[S1] Jensen, K. *et al.* Tcrpmhcmodels: Structural modelling of tcr-pmhc class i complexes. *Scientific Reports* **9**, 1–12 (2019).

[S2] Tadros, D. M., Eggenschwiler, S., Racle, J. & Gfeller, D. The mhc motif atlas: a database of mhc binding specificities and ligands. *Nucleic Acids Research* **51**, D428–D437 (2023).

[S3] Moris, P. *et al.* Current challenges for unseen-epitope tcr interaction prediction and a new perspective derived from image classification. *Briefings in Bioinformatics* **22**, bbaa318 (2021).

[S4] Springer, I., Tickotsky, N. & Louzoun, Y. Contribution of t cell receptor alpha and beta cdr3, mhc typing, v and j genes to peptide binding prediction. *Frontiers in Immunology* **12**, 1436 (2021).
